# Canonical Secretomes, Innate Immune Caspase-1-, 4/11-Gasdermin D Non-Canonical Secretomes and Exosomes May Contribute to Maintain Treg-Ness for Treg Immunosuppression, Tissue Repair and Modulate Anti-Tumor Immunity *via* ROS Pathways

**DOI:** 10.3389/fimmu.2021.678201

**Published:** 2021-05-18

**Authors:** Dong Ni, TingTing Tang, Yifan Lu, Keman Xu, Ying Shao, Fatma Saaoud, Jason Saredy, Lu Liu, Charles Drummer, Yu Sun, Wenhui Hu, Jahaira Lopez-Pastrana, Jin J. Luo, Xiaohua Jiang, Eric T. Choi, Hong Wang, Xiaofeng Yang

**Affiliations:** ^1^ Centers for Cardiovascular Research, Lewis Katz School of Medicine at Temple University, Philadelphia, PA, United States; ^2^ Metabolic Disease Research & Thrombosis Research, Department of Cardiovascular Sciences, Lewis Katz School of Medicine at Temple University, Philadelphia, PA, United States; ^3^ Department of Psychiatry, Lewis Katz School of Medicine at Temple University, Philadelphia, PA, United States; ^4^ Department of Neurology, Lewis Katz School of Medicine at Temple University, Philadelphia, PA, United States; ^5^ Division of Vascular and Endovascular Surgery, Department of Surgery, Lewis Katz School of Medicine at Temple University, Philadelphia, PA, United States; ^6^ Inflammation, Translational & Clinical Lung Research, Lewis Katz School of Medicine at Temple University, Philadelphia, PA, United States

**Keywords:** CD4^+^Foxp3^+^ regulatory T cells (Treg), canonical secretome, innate immune caspase-1 dependent secretome, innate immune caspase-4/11 dependent secretome, immune checkpoint receptors

## Abstract

We performed a transcriptomic analyses using the strategies we pioneered and made the following findings: *1)* Normal lymphoid Tregs, diseased kidney Tregs, splenic Tregs from mice with injured muscle have 3, 17 and 3 specific (S-) pathways, respectively; *2)* Tumor splenic Tregs share 12 pathways with tumor Tregs; tumor splenic Tregs and tumor Tregs have 11 and 8 S-pathways, respectively; *3)* Normal and non-tumor disease Tregs upregulate some of novel 2641 canonical secretomic genes (SGs) with 24 pathways, and tumor Tregs upregulate canonical secretomes with 17 pathways; 4) Normal and non-tumor disease tissue Tregs upregulate some of novel 6560 exosome SGs with 56 exosome SG pathways (ESP), tumor Treg ESP are more focused than other Tregs; 5) Normal, non-tumor diseased Treg and tumor Tregs upregulate some of novel 961 innate immune caspase-1 SGs and 1223 innate immune caspase-4 SGs to fulfill their tissue/SG-specific and shared functions; *6)* Most tissue Treg transcriptomes are controlled by Foxp3; and Tumor Tregs had increased Foxp3 non-collaboration genes with ROS and 17 other pathways; *7)* Immune checkpoint receptor PD-1 does, but CTLA-4 does not, play significant roles in promoting Treg upregulated genes in normal and non-tumor disease tissue Tregs; and tumor splenic and tumor Tregs have certain CTLA-4-, and PD-1-, non-collaboration transcriptomic changes with innate immune dominant pathways; *8)* Tumor Tregs downregulate more immunometabolic and innate immune memory (trained immunity) genes than Tregs from other groups; and *11)* ROS significantly regulate Treg transcriptomes; and ROS-suppressed genes are downregulated more in tumor Tregs than Tregs from other groups. Our results have provided novel insights on the roles of Tregs in normal, injuries, regeneration, tumor conditions and some of canonical and innate immune non-canonical secretomes *via* ROS-regulatory mechanisms and new therapeutic targets for immunosuppression, tissue repair, cardiovascular diseases, chronic kidney disease, autoimmune diseases, transplantation, and cancers.

## Introduction

Immune responses including innate immune macrophages (1), antigen-specific responses (2–13), CD4^+^Foxp3^+^ regulatory T cells (Treg) (14–18) and cosignaling- and immune checkpoint receptors (19) play significant roles in suppressing tumor growth and development. Although cancer immunotherapy is very promising (10, 20), new anti-cancer drugs and vaccines fail to show promising benefits against cancer, which is at least partially due to the infiltration of Treg into the tumor region and suppression of anti-cancer activities of the drugs and vaccines (21). Thus, careful characterizations of Treg in tumor regions and Treg from tumor-bearing models would improve our understanding on transcription changes of Treg in tumor-specific environments (22).

In addition, cardiovascular disease (CVD) risk factors such as hyperhomocysteinemia (23, 24), chronic kidney disease (25–29), hyperlipidemia (30, 31), and hyperglycemia (32, 33) promote vascular inflammation and atherosclerosis *via* several mechanisms. These novel mechanisms include decreased/transdifferentiated Treg (15, 16, 34–36), endothelial cell (EC) function as innate immune cells activation (30, 37–42); caspase-1/inflammasome activation (25, 27, 41, 43), proton leak-regulated mitochondrial reactive oxygen species (ROS) (31, 44, 45); Ly6C^high^ mouse monocyte and CD40^+^ human monocyte differentiation (24, 33, 46, 47); impaired vascular repairability of bone marrow-derived progenitor cells (43, 48); downregulated histone modification enzymes (49) and increased histone 3 lysine 14 acetylation (44), increased expressions of trained immunity pathway enzymes (50, 51) and non-coding RNA regulation (40, 52–54). Due to the significant progress on identification of Treg roles implicated in ischemic injury and repair including myocardial, limb, cerebral ischemia, and apoB autoreactive Treg (55), Treg hold significant therapeutic promise for cardiovascular diseases (56), cardiovascular repair and regeneration (57).

Peripheral differentiations of CD4^+^ T helper cells (Th) result from T cell response to stimulation, *via* innate immune mechanisms (36), by several different inducing cytokines such as interferon-γ (IFN-γ), interleukin-12 (IL-12), and IL-4, and also anatomical locations (58). Naïve CD4+ T cells can be differentiated/polarized into at least nine terminally-differentiated Th cell subsets. These subsets include Th1, Th2, Th9, follicular T (Tfh) (58), Tfh-13 (59), Th17, Treg, Th22 (36, 60), Th25 (61), CD4^+^ cytotoxic T cells (CD4+ CTL) (62), tissue-resident memory T cells (Trm), circulating effector memory T cells (Tem), central memory T cells (Tcm) (63), CD28^null^ T cells (64) as well as other T cell subsets including CD8+ T cells and γδ T cells (64), suggesting that antigen epitopes-independent innate immune inducing cytokine environments play critical roles for naïve Th0 polarization/differentiation into Treg and other Th subsets (16). We previously reported that Treg cell death pathways (17, 34–36, 65–70), Treg generated IL-35 (30, 37, 44, 45, 71–73), and epigenetic pathways (49, 74) may be novel therapeutic targets for maintaining Treg survival (17), preventing immunosuppressive Tregs from becoming pathological Tregs (36), plastic Tregs and even antigen-presenting Tregs (16), and suppressing inflammation (75). Recently, we proposed a novel concept, which suggests that pathological conditions/environments, *via* antigen epitopes-dependent or independent cellular interactions, re-shape physiological Tregs into pathological Tregs that have weakened immuno-suppressive functions and increased plasticity (36). The following supporting evidence published by other investigators validate our proposed model: *First*, Th1-like Treg phenotype (76, 77), and pro-inflammatory IL-17A cytokine secreting Treg (78), which weaken Treg suppression; *Second*, immunosuppression-compromised Treg after myocardial infarction (79); *Third*, four different types of “lymphoma Treg” (15), among which some Treg become malignant; *Fourth*, self-reactive T cells, termed anti-Treg (80), which suppress immune-suppressive cells and secrete pro-inflammatory cytokines; *Fifth*, FOXO3-expressed in tolerogenic dendritic cells (DCs) in modulating Treg and activating anti-Treg (81), and *sixth*, six aspects such as thymic development, peripheral development, homeostasis, function, differentiation, and plasticity of Treg are modulated by 15 cytokines including interleukin-2 (IL-2), IL-15, IL-7, transforming growth factor-β (TGF-β), tumor necrosis factor-α (TNF-α), IL-33, IL-4, IL-1β, IL-6, IL-21, IL-23, IL-12, IL-27, IL-35 (71) and interferon γ (IFNγ) (82, 83), among which proinflammatory cytokines weaken Treg suppression. It is accepted that Tregs undergo phenotypic and functional plastic changes into other Th subsets under pathological conditions (60, 84), which are modulated by co-inhibitory/immune checkpoint receptors (85).

Foxp3 is the major transcription factor (TF), and co-expression of lineage-specifying transcription factors alters the potential function and flexibility of subsets of CD4^+^ T cell; this, in turn, favors the autoimmune pathology (86). Tregs are specialized in the suppression of immuno-pathological reactions in the host immune system against antigens and dangers (36). In addition to inhibition of adaptive immune response, Tregs also play a critical role in controlling various innate immune responses involved in cancers (15), inflammatory diseases including cardiovascular diseases and atherosclerosis (64, 75). However, an important question remained whether Treg have innate immune response machinery. To address this question, we recently reported that Treg secretomes (in lymph nodes and spleen) and transcription factors shared with stem cells contribute to a Treg niche to maintain Treg-ness with 80% innate immune pathways, and functions of immunosuppression and tissue repair (87). Additionally, Tregs play a highly broad spectrum of versatile anti-pathophysiological roles. For example, Tregs facilitate blood flow recovery after ischemia (88), control adipose tissue inflammation, promote muscle repair (89) and maintain tissue/organ homeostasis (90). Treg’s roles in maintaining self-tolerance and prevention of autoimmune responses and chronic inflammation are mediated by various mechanisms including: *a)* Treg killing of target cells (15); *b)* modulation of target cells *via* cell-cell contact; *c)* inhibition of target cells by exosome-carried microRNAs (36); and *d)* secretion of anti-inflammatory/immunosuppressive cytokines (38) including IL-10, IL-35 (30, 71, 72), and TGF-β. Therefore, cellular therapies using Tregs are currently undergoing clinical trials for the treatment of autoimmune diseases, transplant rejection and graft-versus-host disease (91).

In addition to the secretion of anti-inflammatory/immunosuppressive cytokines (38), including IL-10, IL-35 (30, 71, 72), and TGF-β, Treg play a highly tissue- and context-specific roles in angiogenesis either pro- or anti-angiogenic effects (92). Similarly, we recently reported that Treg-secreted IL-35 delays hindlimb ischemia-induced angiogenesis through regulating ROS-extracellular matrix but spares later regenerative angiogenesis (73), indicating that Treg-secreted IL-35 plays roles in a pathological process/phase-specific manner (30, 37, 44, 45, 71–73). Recent progress suggests that Treg secretory proteins could be also much bigger than a list of cytokines and chemokines, since the secretomes from various cell types have been reported (93, 94). The secretome, defined as a portion of total proteins secreted by cells to the extracellular space, secures a proper micro-environmental niche, thus maintaining tissue homeostasis (95, 96). Secreted molecules are key mediators in cell-cell interactions, *via* autocrine, and paracrine manners, and influence the cross-talk with the surrounding tissues in addition to their endocrine functions in long-distance by hormones, growth factors, cytokines, adipokines, myokines, cardiokines (97), and chemokines (98). There is strong evidence supporting that crucial cellular functions such as proliferation, differentiation, communication and migration are regulated strictly by the cell secretome (99). Among the secretomes, we recently reported canonical secretomes of human peripheral blood mononuclear cells (28). Similarly, Treg from mouse spleen, lymph nodes, intestine and visceral adipose tissue may use canonical secretomes (all the human proteins with signal peptide sequence) to fulfill their functions (87). In addition, exosome secretomes, due to their content enriched in proteins, mRNAs and noncoding RNAs, carry out cell-cell communication and promote tissue regeneration, wound healing, extracellular matrix remodeling, immunomodulation (100), angiogenesis, anti-apoptotic activity and cell migration, proliferation and differentiation (101). Moreover, we reported that Treg may use their secretomes shared with stem cells to maintain their Treg-ness with 80% innate immune pathways (87), thus, Treg may also use innate immunity-related secretomes such as caspase-1-gasdermin D (GSDMD) dependent secretome (102), and caspase-4 (humans)/caspase-11 (mice)-GSDMD dependent secretome (103), to carry out their functions. However, it remains unknown that Treg may use canonical secretomes, non-canonical secretomes including exosome secretomes (100), innate immune caspase-1-GSDMD secretomes and innate immune caspase-4/11-GSDMD secretomes for their immune regulatory functions (100).

However, several important knowledge gaps remain: 1) are there differences in Treg transcriptomes between Tregs from normal lymphoid and non-lymphoid tissues, injured tissues, regenerative tissues, tissues from mouse carrying tumor, tumor tissues; 2) are secretomes generated in Tregs in various tissues and pathological conditions; and 3) how Treg transcription factor Foxp3, immune checkpoint receptors cytotoxic T lymphocyte associate protein-4 (CTLA-4, CD152) and programmed cell death-1 (PD-1, CD279) (19), immunometabolic pathways and innate immune memory (trained immunity) and ROS pathways regulate tissue Treg heterogeneity and Treg various secretomes. The major differences between our current study and previous reports on the roles of cytokines and chemokines in Treg are that secretome analyses provide a panoramic view on all the secreted genes in Treg, as opposed to focusing on only one or a few cytokines/chemokines (28).

An overview on the connection logic of the manuscript sections and structure in organizing this manuscript was provided as follows. In the first major part (the Results sections 1, 2, 3, and 4), we compared normal lymphoid tissue Treg, non-tumor diseased kidney Treg and splenic Treg in mouse with injured muscle in the Section 1. In the Section 2, we extended our analysis to examine splenic Treg in mouse with tumors and Treg from tumor tissues. In the Section 3, we summarized all the findings in Treg pathways in normal tissues, non-tumor diseased organs and mouse with injured muscles for comparison. In the Section 4, we identified the differences between tumor Treg and Treg from normal tissues, diseased organs and spleen from the mouse with tumor. Taken together, the sections 1-4 as the first part focused on the transcriptomic heterogeneity of Treg in healthy environments, non-tumor disease conditions and tumors. Treg suppressive functions to inhibit other immune cells depends on several effector mechanisms including secretion of anti-inflammatory/immunosuppressive cytokines such as IL-10, IL-35 and transforming growth factor-β (TGF-β). The second major part in the sections 5, 6 and 7 focused on examining how tissue Treg heterogeneity from normal tissues, non-tumor disease conditions and tumors affect Treg secretory functions; and how Treg secretomes could also potentially affect tissue Treg heterogeneity. We attempted for the first time to introduce all the recent cellular protein secretomic findings to Treg field by examining canonical secretomes with secretory proteins carrying signal peptide, non-canonical secretomes with secretory proteins with no signal peptide such as IL-1β including caspase-1 dependent secretome, caspase-4 dependent secretome and exosome-based secretome. The third major part of this manuscript focused on examining potential molecular mechanisms underlying the Treg heterogeneity and secretomic function differences identified in the first and second major parts from four different angles. These four angles (mechanisms) include Treg-specific transcription factor Foxp3 (nuclear) dependence in the Section 8, major Treg-related immune checkpoint receptors (T cell co-inhibition receptors) on the cell membrane such as PD-1-, CTLA-4- signaling dependence in the Section 9, intracellular immune metabolism/trained immunity (innate immune memory) regulation in the Section 10, and intracellular reactive oxygen species (ROS, a newly reported metabolic sensor system) regulation in the Section 11. Although significant amounts of work in the future are required to verify the -omic data analyses-based findings presented in this paper, similar to other papers with the data generated with -omics such as RNA-sequencing (RNA-Seq) and single cell-RNA-Seq (scRNA-Seq), our results to address the three major questions provide novel insights on tissue Treg heterogeneity in normal, diseased/injured, regenerative, and tumors, Treg secretomes and immunometabolic/trained immunity and ROS regulation of tissue Tregs.

## Materials and Methods

### Expression Profiles of Splenic Treg, LN Treg, Intestine (LP) Treg, and VAT Treg

**Figure 1:**
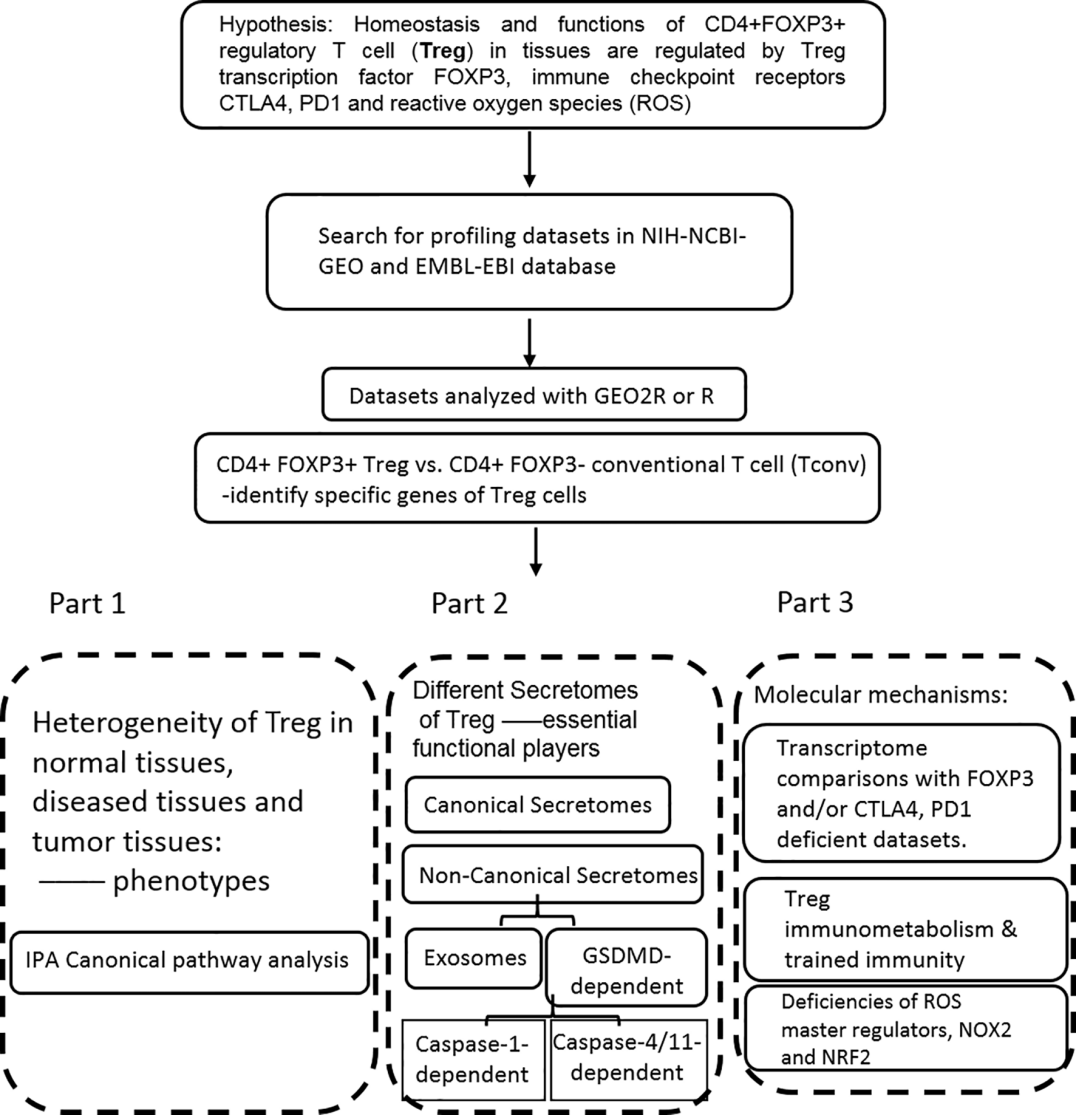
The flow chart of CD4+Foxp3+ regulatory T cell (Treg) transcriptomic and secretomic analyses from normal tissues, diseased tissues and tumor tissues. Two most comprehensive gene expression databases were used in this study: NIH-NIBI-GEO: National Institutes of Health (NIH)-National Center for Biotechnology Information (NCBI)- Gene Expression Omnibus (GEO) databases https://www.ncbi.nlm.nih.gov/gds/; EMBL-EBI: European Molecular Biology Laboratory (EMBL)-European Bioinformatics Institute(EBI) https://www.ebi.ac.uk/arrayexpress/. The gene expression analyses were performed by using the bioinformatic tool/database GEO2R: https://www.ncbi.nlm.nih.gov/geo/geo2r/R: version 3.6.1.

Microarray datasets were collected from National Institutes of Health (NIH)-National Center for Biotechnology Information (NCBI)-Gene Expression Omnibus (GEO) databases (https://www.ncbi.nlm.nih.gov/gds/) and analyzed with an online software GEO2R (https://www.ncbi.nlm.nih.gov/geo/geo2r/). The logic flow of this study was presented in **Figure 1**.

### Statistical Analysis of Microarray Data

We applied a statistical method similar to our previously reported meta-analysis (28, 50, 104). We designed a robust house-keeping gene list (**Supplemental Table 1** of housekeeping genes) with help from Eisenberg and Levanon’s (105) excellent work, including ACTB, GAPDH, PGK1, PPIA, B2M, YWHAZ, SDHA, HMBS, and TBP. Briefly, the mean log fold change (LogFC) of house-keeping genes between treatment and control groups vary from -1.27 to 1.28. The target genes with expression changes more than 2-folds were defined as the upregulated genes, while genes with their expression decreased more than 2-folds were defined as downregulated genes |logFC|>1).

### Ingenuity Pathway Analysis and Metascape Analysis

We utilized Ingenuity Pathway Analysis (IPA, Qiagen, https://www.qiagenbioinformatics.com/products/ingenuity-pathway-analysis/) to characterize clinical relevance and molecular and cellular functions related to the identified genes in our microarray analysis. Differentially expressed genes were identified and uploaded into IPA for analysis. The core and pathways analysis was used to identify molecular and cellular pathways, as we have previously reported (28, 104, 106). For the groups with small numbers of genes modulated, the Metascape analysis (https://metascape.org/gp/index.html#/main/step1) was used for determining signaling pathways involved.

## Results

### Normal Lymphoid Tregs Have Five Specific Pathways, Diseased Kidney Tregs Have 17 Specific Pathways, and Splenic Tregs From Mice With Injured Muscle Have 3 Specific Pathways

**Figure 2:**
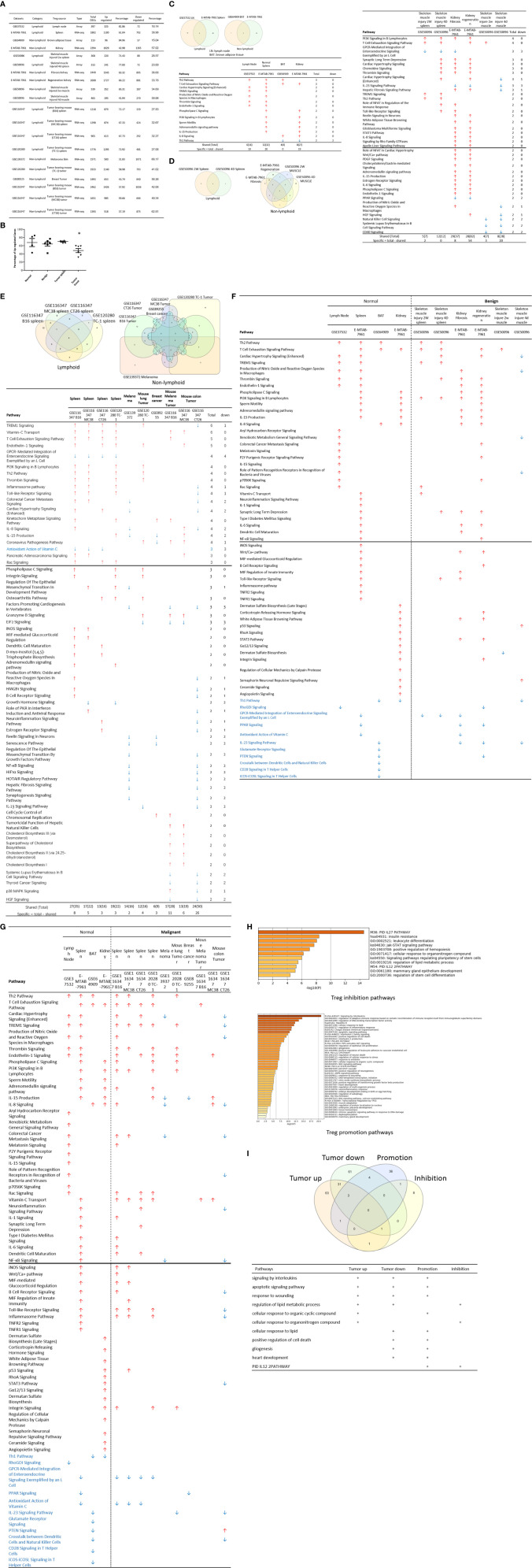
**(A)** Comparisons of regulatory T cells (Treg) versus conventional T cells (Tconv) in tissues enrolled in **Table 1** led to identification of differentially expressed genes (DEGs) in each dataset. The type of results from datasets enrolled including Array data and RNA-seq data. (Cut off: p < 0.05; |Log_2_FC| ≥ 1). **(B)** There were more suppressed DEGs of Treg in Tumor tissues than other 3 groups. The mean percentages of up-regulated genes in tumor tissue Treg groups were 48.22 ± 5.004, lower than that in normal, benign, and tumor spleen groups (67.71 ± 9.806, 64.20 ± 4.891, 70.04 ± 1.458, respectively). *Significantly changed. **(C)** The majority (73.8%, 59/80) of enriched pathways in normal tissue Treg groups were tissue Treg-specific in lymphoid and non-lymphoid tissues. The ingenuity Pathway Analysis (IPA, Qiagen, https://www.qiagenbioinformatics.com/products/ingenuity-pathway-analysis/) using the differentially expressed gene (DEG) from datasets enrolled in this study showed the activated (↑) and inhibited (↓) canonical pathways in normal tissue Treg groups (p < 0.05, |Z-score| >= 2). In addition, Venn diagram analysis was performed to find the commonly shared pathways in this group. The specifically enriched pathways in GSE37532 Lymph node (LN), E-MTAB-7961 spleen, and E-MTAB-7961 Kidney were shown in **Supplementary Table 3A**. In the normal Treg groups, there were a total of 80 pathways enriched including 64 activated and 16 inhibited. There were no dual regulated pathways in normal Treg groups. The most common shared pathway was Th2 Pathway, which was activated in 71.2% (5/7) datasets. TREM1 Signaling, Production of Nitric Oxide and Reactive Oxygen Species in Macrophages, Cardiac Hypertrophy Signaling (Enhanced), and Thrombin Signaling where activated only in Treg from lymphoid tissues. Whereas Th1 Pathway and tRNA Splicing were inhibited, and IL-8 Signaling was activated only in Treg in non-lymphoid tissues. **(D)** Non-lymphoid tissue Treg enriched more specific pathways than lymphoid tissue Treg in benign disease groups. (85 specific pathways in 4 non-lymphoid Treg datasets vs. 2 specific pathways in 2 lymphoid Treg datasets). IPA analysis using the DEG from datasets enrolled in this study showed the activated (↑) and inhibited (↓) canonical pathways in different benign disease tissue Treg groups (p < 0.05, |Z-score| >= 2). Venn diagram analysis was performed to find the commonly shared pathways in this groups. The specifically enriched pathways in various Treg groups were showed in **Supplementary Table 3B**. A total of 124 pathways were enriched in benign disease Treg groups, including 90 activated and 30 inhibited pathways. Cardiac Hypertrophy Signaling (Enhanced), Hepatic Fibrosis Signaling Pathway, Reactive Oxygen Species in Macrophages, and HGF Signaling were dual regulated pathways in non-lymphoid tissue Treg. The percentages of specifically enriched pathways in this group was 70.2% (87/124). 97.7% (85/87) specific pathways were in non-lymphoid tissue Treg datasets. The most commonly shared pathway in benign disease Treg groups were T Cell Exhaustion Signaling Pathway and PI3K Signaling in B Lymphocytes, activating in 67% (4/6) datasets. TREM1 Signaling and Th2 Pathway were only activated in spleen tissue Treg in this group. Whereas IL-23 Signaling Pathway, PPAR Signaling, Natural Killer Cell Signaling, Systemic Lupus Erythematosus In B Cell Signaling Pathway, and CD40 Signaling were inhibited in non-lymphoid tissue Treg. **(E)** Malignant Treg groups enriched more inhibited and dual pathways than normal and benign disease Treg groups. IPA analysis using the DEG from Treg datasets enrolled in this study showed the activated (↑) and inhibited (↓) canonical pathways in different malignant disease tissue Treg groups (p < 0.05, |Z-score| >= 2). Venn diagram analysis was performed to find the commonly shared pathways in this Treg group. The specifically enriched pathways in each dataset were showed in **Supplementary Table 3C**. There were a total of 128 pathways enriched in the malignant Treg groups, including 62 activated, 50 inhibited, and 16 dual regulated pathways. There were 19 specifically enriched pathways in 4 lymphoid Treg datasets (mean 4.8 per dataset), 52 specifically enriched pathways in 6 non-lymphoid Treg datasets (mean 8.7 per dataset). TREM1 Signaling and Vitamin-C Transport were the most commonly shared pathways. In addition, TREM1 Signaling was activated in all the lymphoid tissue Treg and dual regulated in the non-lymphoid tissue Treg. Vitamin-C Transport was activated both in lymphoid and non-lymphoid tissue Treg. **(F)** The Dataset GSE50096 Treg from skeletal muscle injured 4d enriched more down regulated pathways than normal and other benign disease tissue Treg. The pathways enriched in normal lymphoid and non-lymphoid tissue Treg were compared with benign tissue Treg. The 5 pathways all of which were activated in normal tissue Treg were suppressed in Treg from skeletal muscle injured 4d and 2w tissues. All the suppressed pathways in normal tissue Treg groups were also suppressed or non-statistically enriched in benign disease tissue Treg. **(G)** Malignant tissue Treg enriched more down regulated pathways than normal tissue Treg. The Pathways enriched in normal lymphoid and non-lymphoid tissue Tregs were compared with malignant tissue Treg. The 13 pathways all of which were activated in normal tissue Tregs were suppressed in at least one malignant Treg datasets, more specifically, in malignant non-lymphoid tissue Treg. However, only one of the 11 suppressed pathways in normal tissue Treg, PTEN Signaling, was activated in GSE116347 CT26 dataset. **(H)** Metascape analysis of 18 Treg inhibition genes and 57 Treg promotion genes (PMID:32680511) identified 11 Treg inhibition GO and pathways and 45 Treg promotion GO and pathways. Moreover, M54: PID IL12 2PATHWAY was a dual regulated pathway, which was enriched both in Treg promotion and inhibition pathways. **(I)** There were 4 suppressed and 1 activated Treg promotion pathways in malignant Treg datasets whereas there was only 1 activated Treg inhibition pathway in malignant Treg datasets. DEGs in malignant Treg datasets enrolled in this study were analyzed by metascape and compared with Treg promotion and inhibition pathways. Tumor Treg upregulated pathways included up-regulated DEGs in malignant Treg datasets whereas tumor downregulated pathways included down-regulated DEGs in malignant Treg datasets. The 4 downregulated Treg promotion pathways were cellular response to lipid, positive regulation of cell death gliogenesis, gliogenesis, and heart development. One up-regulated Treg inhibition pathway was cellular response to organonitrogen compound. The 61 Tumor downregulated, 31 dual, and 63 tumor upregulated pathways were summarized in **Supplementary Table 4**.

We recently reported that Treg-specific transcription factor Foxp3 is expressed in trachea, thymus, spleen, mammary gland, lymph node, lung, eye, and blood (16). Six aspects involved in development, homeostasis, function, differentiation, and plasticity of Treg are modulated by 15 cytokines including IL-2, IL-15, IL-7, TGF-β, TNF-α, IL-33, IL-4, IL-1b, IL-6, IL-21, IL-23, IL-12, IL-27, IL-35 (71) and IFNγ (82). Since these cytokine expression levels are varied among tissues, thus, various Treg populations in these tissues have been identified (90), which are similar to what we reported on tissue macrophages that 20 novel disease group-specific and 12 new shared macrophage pathways have been found in eight groups of 34 diseases including 24 inflammatory organ diseases and 10 types of tumors (1). We hypothesized that tissue Treg have different signaling pathways and Treg from non-malignant diseases have also different signaling pathways. To test this hypothesis, we collected 20 Treg microarrays and RNA-Seq datasets from two comprehensive databases such as NIH-NCBI-Geo Datasets database (https://www.ncbi.nlm.nih.gov/geo/) and EMBL-EBI Arrayexpress (https://www.ebi.ac.uk/arrayexpress/). As shown in **Table 1**, 20 datasets included one from spleen, one from lymph nodes (LN), one from brown adipose tissue (BAT), one from kidney, two from spleens in mouse with injured skeletal muscles for four days and two weeks, respectively, two from mouse injured skeletal muscles for four days and two weeks, respectively, one from kidney fibrosis, one from kidney regeneration, four from spleens of tumor bearing mouse (BL6, MC38, CT26 and TC-1), respectively, seven tumor tissue Treg from hepatocellular carcinoma, melanoma, lung tumor (TC-1), breast cancer, tumor bearing Bl6, tumor bearing MC38, and tumor bearing CT26, respectively. Of note, As the original paper reported (107), tumor infiltrating Treg and tumor splenic Treg were sorted as live, CD45+TCRb+CD4+Foxp3+CD8-DUMP-). Tumor Treg were distinguished from tumor splenic Treg using RNA-Seq analysis (107). In addition, one of our recent manuscripts mainly focused on profile on Treg transcription markers, CD markers, kinome, of tissue Treg from mouse spleen, lymph nodes, small intestines and white adipose tissues, which are not overlapped in this study (87). As shown in **Figure 2A** two third of the datasets were collected from RNA-Seq, suggesting that new techniques were used. Total differentially expressed genes (DEGs) were varied among Treg datasets, ranging from 113 (brown adipose tissue Treg) to 2608 (regeneration kidney Treg). The upregulated genes in total DEGs among Treg datasets were ranging from 31.8% (melanoma skin Treg) to 85.0% (brown adipose tissue Treg). As shown in **Figure 2B**, there were more downregulated genes in Treg from tumor tissue groups than normal tissue Treg, diseased tissue Treg groups and tumor splenic Treg groups.

**Table 1:** Twenty-three Treg transcriptomic datasets collected from the NIH-NCBI-GEO and EMBL-EBI databases were analyzed.

Access ID	Group	Tissue	Comparison	PMID
GSE37532	normal lymph node	lymph node	CD3+CD4+CD25+ Treg cells vs. CD3+CD4+CD25- Tconv	25550516
E-MTAB-7961	normal spleen	Spleen	CD45+ CD4+ TCRB+ Foxp3+ Treg vs CD45+ CD4+ TCRB+ Foxp3- Tconv	32051345
GSE64909	normal tissue	Brown adipose tissue(BAT)	CD4+CD25+Foxp3+ Treg vs CD4+Foxp3- Tconv	25714366
E-MTAB-7961	normal tissue	Kidney	CD45+ CD4+ TCRB+ Foxp3+ Treg vs CD45+ CD4+ TCRB+ Foxp3- Tconv	32051345
GSE50096	benign disease spleen	Skeletal muscle injured 2w spleen	CD4+ Foxp3+ Treg vs CD4+ Foxp3- Tconv	24315098
GSE50096	benign disease spleen	Skeletal muscle injured 4d spleen	CD4+ Foxp3+ Treg vs CD4+ Foxp3- Tconv	24315098
E-MTAB-7961	benign disease tissue	Kidney Fibrosis	CD45+ CD4+ TCRB+ Foxp3+ Treg vs CD45+ CD4+ TCRB+ Foxp3- Tconv	32051345
E-MTAB-7961	benign disease tissue	Kidney Regeneration	CD45+ CD4+ TCRB+ Foxp3+ Treg vs CD45+ CD4+ TCRB+ Foxp3- Tconv	32051345
GSE50096	benign disease tissue	Skeletal muscle injured 2w muscle	CD4+ Foxp3+ Treg vs CD4+ Foxp3- Tconv	24315098
GSE50096	benign disease tissue	Skeletal muscle injured 4d muscle	CD4+ Foxp3+ Treg vs CD4+ Foxp3- Tconv	24315098
GSE116347	tumor spleen	Tumor Bearing Mouse (B16)	CD4+Foxp3+ Treg vs CD4+Foxp3- Tconv	30348759
GSE116347	tumor spleen	Tumor Bearing Mouse (MC38)	CD4+Foxp3+ Treg vs CD4+Foxp3- Tconv	30348759
GSE116347	tumor spleen	mTumor Bearing Mouse (CT26)	CD4+Foxp3+ Treg vs CD4+Foxp3- Tconv	30348759
GSE120280	tumor spleen	Lung Tumor(TC-1) Bearing Mouse	CD4+Foxp3+ Treg vs CD4+Foxp3- Tconv	NA
GSE103523*	tumor tissue	Hepatocellular carcinoma	CD14-CD4+CD25high Treg cells vs. CD3+CD4+CD25- Tconv cells	29941600
GSE139372	tumor tissue	Melanoma	CD4+CD8- CD25+CD27+ Treg vs CD4+CD8- CD25-CD27- Teff	31852848
GSE120280	tumor tissue	Lung Tumor(TC-1) Bearing Mouse	CD4+Foxp3+ Treg vs CD4+Foxp3- Tconv	NA
GSE89225	tumor tissue	Breast Cancer	CD4+Foxp3+ Treg vs CD4+Foxp3- Tconv	27851913
GSE116347	tumor tissue	Tumor Bearing Mouse (B16)	CD4+Foxp3+ Treg vs CD4+Foxp3- Tconv	30348759
GSE116347	tumor tissue	Tumor Bearing Mouse (MC38)	CD4+Foxp3+ Treg vs CD4+Foxp3- Tconv	30348759
GSE116347	tumor tissue	Tumor Bearing Mouse (CT26)	CD4+Foxp3+ Treg vs CD4+Foxp3- Tconv	30348759

NIH-NIBI-GEO, National Institutes of Health (NIH)-National Center for Biotechnology Information (NCBI)- Gene Expression Omnibus (GEO) databases https://www.ncbi.nlm.nih.gov/gds/; EMBL-EBI, European Molecular Biology Laboratory (EMBL)-European Bioinformatics Institute(EBI) https://www.ebi.ac.uk/arrayexpress/; Mouse models involved in this study were described in supplementary table 2. Access ID start with “GSE” was a dataset from GEO database, whereas start with “E-MTAB” was a dataset from EMBL-EBI dataset. GFP, green fluorescence protein; TCRB, T cell receptor beta locus. NA, Not Available.

*Up to August 14th, 2020, a total of 7 datasets of Treg vs Tconv in malignant tumor tissue were found in the GEO database (https://www.ncbi.nlm.nih.gov/geo/). GSE103523 was excluded because Foxp3 was not a significantly DEG in this dataset. The differentially expressed genes (DEGs) of Treg versus (vs) CD4+Foxp3- T effector cells were screened as a p < 0.05 and absolute |log2FC|>= 1. The datasets were grouped as normal, benign disease and malignant Lymphoid and non-lymphoid tissues.

The Ingenuity Pathway Analyses were performed based on the DEGs in Treg in comparison to that of CD4^+^Foxp3^-^ T effector cells (Teff). As shown in **Figure 2C**, Treg from lymphoid tissues, LN and spleen, shared six pathways including Th2 pathway, cardiac hypertrophy signaling, TREM1 signaling, nitric oxide and reactive oxygen species, thrombin signaling and endothelin signaling. Treg from BAT (108) and kidney shared upregulation of two pathways, including T cell exhaustion signaling and IL-8 signaling, and downregulation of Th1 pathway. Of note, it has been reported that visceral adipose tissue (VAT) Treg are Foxp3^+^ peroxisome proliferator-activated receptor-γ (PPARγ)^+^ Treg with increased lipid metabolism (109), which are enhanced by IL33-ST2 axis (110); and among 194 significantly upregulated genes in BAT Treg, 148 genes are identified as VAT Treg-specific, whereas only 10 genes are BAT Treg-specific (108). Interestingly, Treg from spleen shared five pathways with kidney, such as phospholipase C signaling, phosphoinositide 3-kinase (PI3K) signaling, sperm motility, adrenomedullin signaling, and IL-15 production. As shown in **Figure 2D**, Treg from the spleens of mouse with skeletal muscle injury 2 weeks, the spleen of mouse with skeletal muscle injury 4 days, kidney fibrosis, kidney regeneration shared PI3K signaling. Treg from the spleens of mouse with skeletal muscle injury 2 weeks, the spleen of mouse with skeletal muscle injury 4 days, kidney regeneration, and mouse skeletal muscle injury 2 weeks shared T cell exhaustion signaling. Treg from the spleen of mouse with skeletal muscle injury 4 days, kidney regeneration shared four pathways such as synaptic long term depression, cardiac hypertrophy, chemokine signaling and thrombin signaling. Two pathways such as TREM1 signaling and Th2 pathway showed in Treg from the spleens of mouse with skeletal muscle injury 2 weeks, and the spleen of mouse with skeletal muscle injury 4 days. Of note, it has been reported that skeletal muscle Treg promote skeletal muscle regeneration by producing amphiregulin (Areg), to member of the epidermal growth factor family whose receptor is expressed on satellite cells in the skeletal muscles (110). Other two pathways such as role of NFAT and Toll-like receptor signaling showed in Treg from the spleens of mouse with skeletal muscle injury 4 days and kidney fibrosis. Of note, Treg play significant roles in maintaining normal kidney and suppressing acute ischemic kidney injury, preconditioning prevention of ischemia reperfusion injury, inhibition of cisplatin-induced renal injury and adrianmycin nephropathy, suppression of autoimmune glomerulopathies, lupus glomerulonephritis, nephrotoxic nephritis and diabetic kidney disease (111). In addition, 17 pathways were shared by Treg from kidney fibrosis and kidney regeneration. These kidney Treg-specific pathways include: reelin signaling, white adipose tissue browning, glioblastoma multiform, STAT3, IL-8, Rho GTPases, apelin liver, role of NFAT in cardiac hypertrophy, Wnt/Ca2^+^, PDGF, cholecystokinin/gastrin, adrenomedullin, IL-15 production, estrogen receptor, IL-6, phospholipase C and endothelin-1. Moreover, three pathways including natural killer cells, systemic lupus and CD40 signaling were downregulated in Treg from skeletal muscle injury 2 weeks and skeletal muscle injury 4 days, which were muscle injury Treg-specific. Of note, although tissue Treg heterogeneity has been identified, Treg-specific signatures remain including Treg markers such as CD4+Foxp3+. These common Treg signatures allow us to study the effects of transcriptomic differences of tissue Treg, Treg from diseased conditions, tumor environments and tumor-carrying mouse on secretomic pathways as well as Treg niche-secretomes interactions. Similar approaches were reported by other investigators (110, 112). Taken together, Treg from normal lymphoid tissues such as lymph nodes (LN) and spleen have five specific pathways, Treg from brown adipose tissue have no specific pathways, Treg from kidney fibrosis and kidney regeneration have 17 specific pathways, and Treg from spleen of mouse with injured skeletal muscle have three muscle injury-specific pathways.

### Tumor Splenic Tregs Share 12 Pathways With Tumor Tregs; Tumor Splenic Tregs Have 11 Specific Pathways; and Tumor Tregs Have 8 Specific Pathways.

We then hypothesized that Treg from spleens of mouse bearing tumors and Treg isolated from tumor tissues have specific signaling pathways. To test this hypothesis, the Ingenuity Pathway Analyses (IPA) were performed based on the DEGs in Treg in comparison to that of CD4^+^Foxp3^-^ T effector cells (Teff). As shown in **Figure 2E**, 12 pathways were shared by Treg from spleens of mouse bearing tumors and Treg from tumor tissues including TREM1, Vitamin C transport, T cell exhaustion, endothelin-1, PI3K signaling, Th2 pathway, thrombin, kinetochore metaphase, pancreatic adenocarcinoma, phospholipase C, integrin, and osteoarthritis. Of note, the sources of Tregs were specified in **Figure 2E** and were also detailed in **Table 1**. Eleven pathways were tumor-bearing spleen Treg-specific including inflammasome, Toll-like receptor, colorectal cancer metastasis, cardiac hypertrophy, Rac, epithelial-mesenchymal transition, iNOS, MIF-mediated glucocorticoid regulation, dendritic cell maturation, inositol (1,4,5)-triphosphate biosynthesis, and adrenomedullin. Treg from tumor tissues had eight specific pathways including coronavirus pathogenesis, granzyme B, cell cycle control, Natural killer cell, cholesterol biosynthesis III, superpathway of cholesterol biosynthesis, cholesterol biosynthesis II and cholesterol biosynthesis I.

Taken together, our results have demonstrated that *first*, Treg from spleens of mouse bearing tumors share 12 pathways with Treg from tumor tissues; *second*, Treg from spleens of mouse bearing tumors have 11 specific pathways, suggesting that tumorigenesis affect distal splenic Treg significantly; and *third*, Treg from tumor tissues have eight specific pathways. Our new findings are correlated with previous reports that Treg compartmentalization and trafficking may be tissue or/and organ specific, in which distinct chemokine receptor and integrin expression may contribute to selective retention and trafficking of Treg cells to sites where regulation is required (113). These Treg trafficking receptors include chemokine receptors and other G-protein coupled receptors, integrins, as well as selectins and their ligands. These receptors enable Treg to: *1)* enter appropriate tissues from the bloodstream *via* post-capillary venules, *2)* navigate these tissues to locally execute their immune-regulatory functions, and *3)* seek out the right antigen-presenting cells to interact with in order to receive the signals that sustain their survival, proliferation, and functional activity (114).

### 52 Pathways Are Upregulated and 11 Pathways Are Downregulated in Tregs From Normal Tissues, and Disease Tissues

We hypothesized that Treg from normal tissues and non-malignant disease tissues have shared upregulated pathways and downregulated pathways. As shown in **Figure 2F**, 52 upregulated pathways were shared by Treg from normal tissues and non-malignant disease tissues and 11 pathways were downregulated in Treg from normal tissues and non-malignant disease tissues including Th1 pathway, RhoGDI signaling, GPCR-mediated integration of enteroendocrine signaling, PPAR signaling, antioxidant action of vitamin C, IL-23 signaling, glutamate receptor, PTEN, cross-talk between dendritic cells and natural killer cells, CD28 signaling, and iCOS-iCOSL signaling. Of note, the sources of Tregs were specified in **Figure 2F** and were also detailed in **Table 1**. Previous report showed that systemic ablation of Treg compromises the adaptation of whole body energy expenditure to cold exposure, correlating with impairment in thermogenic gene expression and massive invasion of proinflammatory macrophages in BAT (108). The four pathways were downregulated only in Treg from BAT including glutamate receptor, cross-talk between dendritic cells and natural killer cells, CD28 signaling, and iCOS-iCOSL signaling. Our results may suggest that *first*, thermogenic environment in BAT may play significant roles in downregulating those four pathways; and *second*, these pathway downregulation in BAT Treg may play important roles.

We then hypothesized that Treg from injured tissues have pathway changes from that of normal tissues. To examine this hypothesis, we compared four datasets from Treg in normal tissues such as LN, spleen, BAT and kidney to that of six Treg datasets including spleen Treg of mouse with injured muscles for 2 weeks and Treg from mouse with injured muscles for four days, kidney fibrosis, kidney regeneration, Treg from injured muscle for two weeks and Treg from injured muscle for four days. As shown in **Figure 2F**, five pathways were upregulated in normal tissues but downregulated in injured muscles including cardiac hypertrophy signaling (enhanced), production of nitric oxide and reactive oxygen species, xenobiotic metabolism signaling, role of pattern recognition receptors, and dermatan sulfate biosynthesis.

Taken together, our results have demonstrated that fifty two pathways are upregulated in Treg from normal lymphoid, non-lymphoid tissues and non-malignant diseases; eleven pathways are downregulated in Treg from normal lymphoid, non-lymphoid tissues and non-malignant diseases; four pathways are downregulated only in Treg from BAT; and five pathways upregulated in Treg from normal tissues are downregulated in injured muscles.

### Upregulated Genes in Tumor Tregs Are Lower Than That in Tregs From Normal Tissues, Diseased Tissues, and Tumor Spleens; Tumor Tregs Are Different From Tregs From Normal, Diseased Tissues and Tumor Spleens in 11 New Innate Immune Pathways

It has been reported that Treg depletion alters the tumor microenvironment and accelerates pancreatic carcinogenesis (115), suggesting that Treg play critical roles in controlling tumor growth. We hypothesized that Treg transcriptomes from normal tissues are different from that of Treg from tumor tissues; and some pathways in Treg from normal tissues are different from that in malignant tissues (116). As shown in **Figure 2B**, upregulated gene numbers in Treg from tumor tissues in comparison to that Teff cells in the same tissues were significantly lower than that of Treg from normal tissues, non-malignant diseases and spleens of mouse with tumors. These results have demonstrated that Treg transcriptomes undergo significant remodeling in tumor tissues.

As shown in **Figure 2G**, 13 pathways upregulated in normal tissue Treg were downregulated in Treg from malignant tissues including cardiac hypertrophy signaling (Enhanced), TREM-1 signaling, production of nitric oxide and reactive oxygen species in macrophages, IL-15 signaling, IL-8 signaling, colorectal cancer metastasis signaling, role of pattern recognition receptors in recognition of bacteria and viruses, neuroinflammation signaling pathway, NF-kB signaling, B cell receptor signaling, Toll-like receptor signaling, inflammasome pathway, and STAT3 pathway. Of note, the sources of Tregs were specified in **Figure 2G** and were also detailed in **Table 1**. It has been reported that 18 regulators are downregulated in Treg in tumors including GATA3, STAT3, STAT4, RORγ, IL-6, IL-17, IL-1β, DNMT3a, Stub1, Id2, E47, Spi-B, SOCS3, AKT, mTORC1, Raptor, and Glut1 (116). In addition, one pathway, PTEN signaling, was downregulated in normal tissue Treg and upregulated in Treg from mouse colon tumor. Of note, 13 out of 14 pathways are innate immune pathways, suggesting that innate immune pathways play significant roles in maintaining Treg suppressive functions. As recent review reported several pathways in Treg from cancers including Foxp3, IRF4, PI3/AKT/FoxO axis, Helios, Eos, Nr4a, Bach2, E proteins and their inhibitor of DNA-binding (Id) counterparts, STAT3, and NF-kB (22). In addition, it has been reported that 57 regulators are promoted in Treg in tumors, including CCL2, CCR2, IL-2, IL-27, IL-33, IL-10, TGF-β, NFAT, SMAD3/4, AP1, PP2A, TLR8, Id3, Nr4a, CD36, ST2, YAP, Mst1/2, KAP1, TRAF3IP3, CnB, NLK, Tram, Neuropilin-1, Calcineurin, TAK1, VHL, C-Rel, NF-kBp65, RelA, P100, PI3Kp110z, IKKb, CREB, CBP/p300, STAT5, TRAF6, TNFR2, AMBRA1, HIC1, HIF1-HRF8, Demethylated TSDR, EZH2, SENP3, ATF-CNS2, C-RelCNS3, Blimp1, MALT1, LKB1, mTORC2, Rictor, Foxp1, Foxo3, Foxp3, Helios, Hypoxia, and Fatty acid (116). Using IPA analysis, our results have identified 11 new signaling pathways including cardiac hypertrophy signaling (Enhanced), TREM-1 signaling, production of nitric oxide and reactive oxygen species in macrophages, IL-15 signaling, IL-8 signaling, colorectal cancer metastasis signaling, role of pattern recognition receptors in recognition of bacteria and viruses, neuroinflammation signaling pathway, B cell receptor signaling, Toll-like receptor signaling, and inflammasome pathway. Of note, due to missing expression data of reported Treg genes, we could not use IPA for the pathway analysis. As shown in **Figure 2H**, by using the Metascape pathway analysis, we found 45 pathways associated with 57 reported Treg upregulated genes and 11 pathways associated with 18 reported Treg downregulated genes (116). For the comparison, we also re-analyzed the pathways of tumor Tregs in our study with the Metascape pathway analysis. As shown in **Figure 2I**, 100 upregulated pathways were found for tumor Treg, 100 downregulated pathways were found for tumor Treg and 35 dual pathways were found to be shared by upregulated pathways of tumor Treg and downregulated pathways of tumor Treg. Additional Venn Diagram analysis showed that by comparison of our Metascape pathway data on tumor Treg to that of reported tumor Treg, our results have demonstrated 63 new upregulated tumor Treg pathways, 61 new downregulated tumor Treg pathways and 31 new dual pathways shared by upregulated tumor Treg pathways and downregulated tumor Treg pathways.

Taken together, our results have demonstrated that Treg from tumors are different from Treg from normal tissues, non-malignant disease tissues and spleens of mouse with tumors in thirteen innate immune IPA pathways and one adaptive immune IPA pathway. In addition, we have also identified 63 new tumor Treg upregulated Metascape pathways, 61 new tumor Treg downregulated Metascape pathways, and 31 new dual Metascape pathways. Moreover, we found that four Metascape pathways including signaling by interleukins, apoptotic signaling pathway, response to wounding, and regulation of lipid metabolic process are shared by three groups of tumor Treg pathways and seven Metascape pathways are shared by two groups of tumor Treg pathways. These results suggest that tumor microenvironments modulate Treg transcriptomes in specific manners; shared pathways may be tumor Treg essential pathways; and tumors modulate Treg and suppress anti-tumor immune responses and immunosurveillance mechanisms as we and other reported (15).

### Normal and Non-Tumor Disease Tregs Upregulate Canonical Secretomic Genes With 24 Pathways, Share Pathways Such as P38 MAPK, TLR/IL-1, and IL-6 Pathways; and Tumor Tregs Upregulate Canonical Secretomic Genes in 17 Pathways With Tumor-Treg Specific Manners

**Figure 3:**
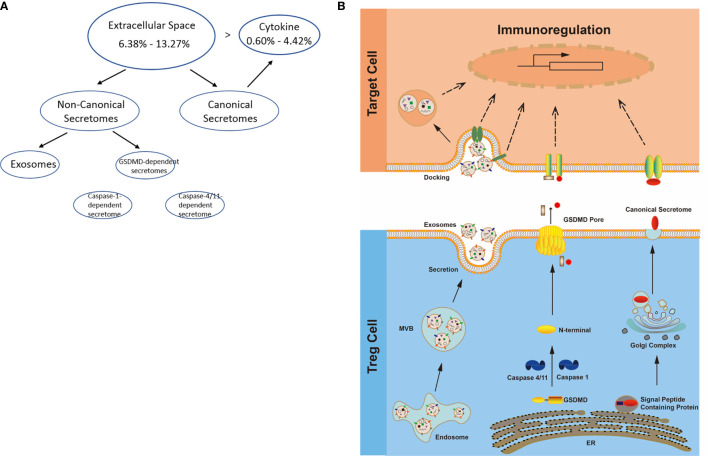
**(A)** Several secretomic pathways of Treg may contribute to the compositions of extracellular space molecules from Treg. In all the datasets, the percentages of extracellular space molecules were varied from 6.38 to 13.27%, which were much higher than the percentages of cytokines. Therefore, numerous additional non-canonical secretome molecules were existed in the extracellular space. The mechanisms of canonical and non-canonical secretome transportation were shown in **Figure 1B**. **(B)** The secretion mechanisms such as canonical (proteins with signal peptide) and non-canonical secretomes (proteins with no signal peptide) may contribute to the Treg secretory molecule repertoire. The proteins in canonical secretory pathway are secreted *via* endoplasmic reticulum (ER)-Golgi complexes-plasma membrane pathway (right in the lower panel). In contrast, proteins having no signal peptide are secreted *via* caspase-1-gasdermin D (GSDMD) pore/channel-dependent pathway (center in the lower panel), caspase-4 (humans)/11 (mice)-GSDMD-dependent pathway (center in the lower panel) and exosome pathway (left in the lower panel). Treg may use these secretomic proteins (including all the cytokines and chemokines) to fulfill their immunoregulatory functions by binding to their receptors or docking on exosome up-taking mechanisms on the plasma membrane.

**Figure 4:**
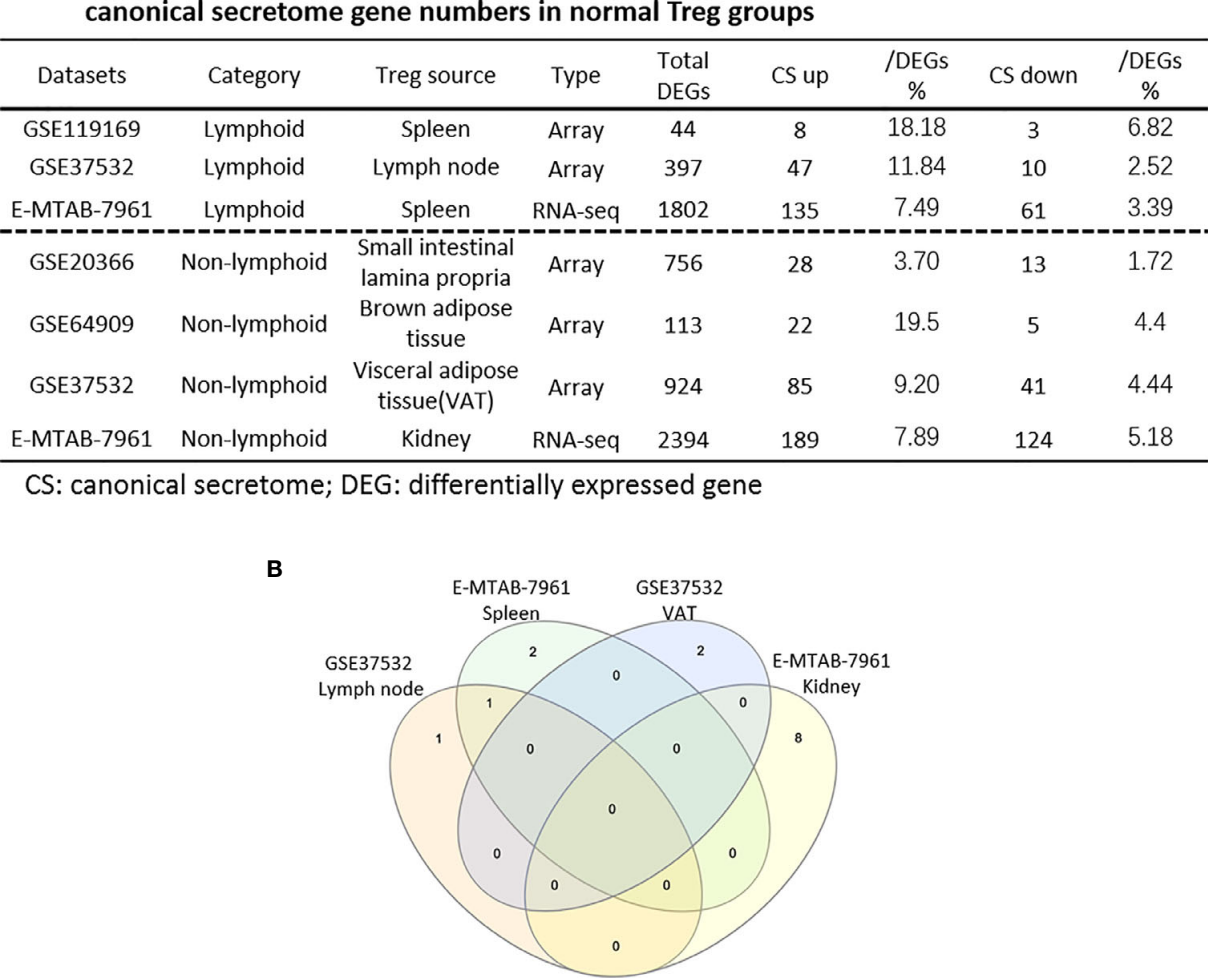
**(A)** The majority (13/14) of canonical secretome pathways in normal Treg groups were specific in lymphoid and non-lymphoid tissue Treg. A list of human 2640 canonical secretome (CS) genes were collected from our previous report (PMID: 32179051). The pathways associated with the DEGs in the Treg canonical secretome in each datasets were analyzed by IPA. **(B)** A total of 14 canonical secretome pathways were enriched in the normal Treg groups. There were no pathways enriched in GSE119169 spleen Treg, GSE20366 Small intestinal lamina propria Treg, and GSE64909 brown adipose tissue Treg. GSE37532 Lymph node Treg and E-MTAB-7961 spleen Treg shared a common p38 MAPK Signaling pathway. LPS/IL-1 Mediated Inhibition of RXR Function was enriched in GSE37532 lymph node Treg. IL-6 Signaling and Type I Diabetes Mellitus Signaling were enriched in E-MTAB-7961 spleen Treg. Toll-like Receptor Signaling and Leukocyte Extravasation Signaling were enriched in GSE37532 visceral adipose tissue (VAT) Treg. VEGF Family Ligand-Receptor Interactions, Nitric Oxide Signaling in the Cardiovascular System, VEGF Signaling, eNOS Signaling, White Adipose Tissue Browning Pathway, Ovarian Cancer Signaling, and BEX2 Signaling Pathway were enriched in E-MTAB-7961 Kidney tissue Treg. These specifically enriched canonical secretome pathways indicate that Treg cells in different tissues have diverse secreting functions. (cut off: p < 0.05 and |Z-score| >= 2).

It has been reported that IL-2 is important for Treg survival (17), IL-2 and TGF-β are critical for Treg induction (16), IL-33/ST2 axis is important for VAT Treg (109) and IL-35 is essential for iTreg 35 induction (36, 71, 117, 118). In addition, IL-37 is highly expressed in Treg of patients with melanoma and enhanced by melanoma secretome. However, it remains poorly understood that Treg secretomic changes in normal tissue Treg, disease tissue Treg and tumor Treg. It has been reported that the Nod-like receptor family 3 (NLRP3) promotes gastro-intestinal CD11b^+^ dendritic cell differentiation and Treg response in inflammasome-independent manner (119). In contrast, NLRP3 has also been reported to inhibits the expression of Foxp3 independent of inflammasome activation in Tregs. NLRP3- deficient mice elevate Treg generation in various tissues in the *de novo* pathway. NLRP3 deficiency increases the amount and suppressive activity of Treg populations, whereas NLRP3 overexpression decreases Foxp3 expression and Treg levels (120).The inflammatory cell death (pyroptosis) (121) carried out by caspase-1 (122) canonical, caspase-4 (humans)/caspase-11 (mice) non-canonical inflammasomes-Gasdermin D (123) pathway has been reported to mediate T cell death (124–127). Due to their content enriched in proteins, mRNAs and noncoding RNAs, exosome secretomes carry out cell-cell communication and promote tissue regeneration, wound healing, extracellular matrix remodeling, immunomodulation (100), angiogenesis, anti-apoptotic activity and cell migration, proliferation and differentiation (101). However, it remains unknown that Treg use caspase-1-Gasdermin D secretome, caspase-4-Gasdermin D secretome and exosome secretomes (100). We hypothesized that the expressions of Treg canonical secretome, three types of non-canonical secretomes such as caspase-1-Gasdermin D, caspase-4-Gasdermin D and exosome secretome are modulated in normal tissues, diseases and tumors. To examine this hypothesis, as shown in **Figures 3A, B**, we collected four types of secretomes with total 11,385 proteins including canonical secretome (signal peptide-mediated exocytic secretory pathway; 2,641 proteins) (28), caspase-1-dependent non-canonical secretome (non-signal peptide-mediated;961 proteins) (102), caspase-4-dependent non-canonical secretome (1,223 proteins) (103), and exosome secretome (6,560 proteins, downloaded from a comprehensive exosome database http://exocarta.org/download) (100). As shown in **Figure 4A**, canonical secretomic genes were upregulated in splenic Treg (8 genes), LN Treg (47 genes), splenic Treg (RNA-Seq, 135 genes), small intestine Treg (28 genes), BAT Treg (22 genes), VAT Treg (85 genes) and kidney Treg (189 genes), respectively. Of note, the sources of Tregs were specified in the **Figure 4** and were also detailed in **Table 1**. We then performed the IPA for signaling pathways that upregulated secretomic genes are involved in. A total of 14 canonical secretome pathways were enriched in the normal Treg group (cut off: *p* < 0.05 and |Z-score| >= 2). There were no pathways enriched in splenic Treg (GSE119169) (not shown). As shown in **Figure 4B**, small intestine Treg, and BAT Treg. LPS/IL-1 mediated inhibition of RXR function was enriched in LN Treg. IL-6 signaling and type I diabetes mellitus signaling were enriched in splenic Treg (E-MTAB-7961). Toll-like receptor signaling and leukocyte extravasation signaling were enriched in VAT Treg. LN Treg and splenic Treg (E-MTAB-7961) shared a common p38 MAPK signaling pathway. Seven pathways such as VEGF family ligand-receptor interactions, nitric oxide signaling in the cardiovascular system, VEGF signaling, eNOS signaling, white adipose tissue browning pathway, ovarian cancer signaling, and BEX2 signaling pathway were enriched in kidney Treg (E-MTAB-7961). Taken together, these results have demonstrated that *1)* lymphoid tissue Treg secretomes carry four inflammation-related functions such LPS/IL-1, IL6 signaling, type I diabetes signaling and p38 MAPK signaling; *2)* VAT Treg play significant roles in Toll-like receptor signaling and leukocyte extravasation signaling; and *3)* regeneration kidney Treg secretomic genes play critical roles in promoting kidney regeneration.

As shown in **Table 2A**, canonical secretomic genes were upregulated in splenic Tregs from skeletal muscle injured for 2 weeks (48 genes) and skeletal muscle injured for 4 days (47 genes), fibrosis kidney (179 genes), regeneration kidney (265 genes), Treg from skeletal muscle injured for 2 weeks (74 genes), and Treg from skeletal muscle injured for 4 days (49 genes), respectively. Of note, the sources of Tregs were specified in the **Table 2A** and were also detailed in **Table 1**. These results showed that splenic Treg from injured skeletal muscles increased more canonical secretomic genes compared to normal splenic Treg (**Figure 4**); Treg from injured skeletal muscles (2 weeks) increased more canonical secretomic genes compared to that of Treg from injured skeletal muscles (4 days); Treg from regeneration kidney increased more canonical secretomic genes compared to that of Treg fibrosis kidney. In the benign disease Treg group as shown in **Table 2B**, a total of 24 activated pathways were enriched in upregulated secretomic genes. P38 MAPK signaling were shared in five Treg groups including splenic Treg (injured muscle for 2 weeks), splenicTreg (injured muscle for 4 days), skeletal muscle Treg (injured muscle for 2 weeks), skeletal muscle Treg (injured for 4 days), and Treg from fibrosis kidney. STAT3 pathway was shared in two kidney Treg sets. IL-6 signaling was increased in skeletal muscle Treg (injured for 4 days). Four of the canonical secretome pathways were specific in E-MTAB-7961 Treg from kidney regeneration dataset, including hepatic fibrosis signaling pathway, PDGF signaling, VEGF signaling, and VEGF family ligand-receptor interactions, which may carry functions of tissue repair. IL-6 signaling was specifically enriched in GSE50096 Treg from muscle injury 4 days dataset, which was in the acute reaction phase after muscle injury.

Table 2AAll the specific canonical secretome pathways were enriched in the E-MTAB-7961 kidney regeneration Treg. A. Canonical secretome gene numbers in benign disease Treg groups.

**Table:** 

Datasets	Category	Treg source	Type	TotalDEGs	CS up	/DEGs%	CS down	/DEGs%
GSE50096	Lymphoid	Skeletal muscle injured 2w spleen	Array	308	48	15.58	14	4.55
GSE50096	Lymphoid	Skeletal muscle injured 4d spleen	Array	313	47	15.02	12	3.83
E-MTAB-7961	Non-lymphoid	Fibrosis kidney	RNA-seq	2440	179	7.34	71	2.91
E-MTAB-7961	Non-lymphoid	Regeneration kidney	RNA-seq	2608	265	10.16	86	3.30
GSE50096	Non-lymphoid	Skeletal muscle injured 2w muscle	Array	539	74	13.73	27	5.01
GSE50096	Non-lymphoid	Skeletal muscle injured 4d muscle	Array	465	49	10.54	39	8.39

CS, canonical secretome; DEG, differentially expressed gene. IPA analysis was performed to identify upregulated canonical secretome pathways in benign disease tissues (cut off: p < 0.05 and |Z-score| >= 2). In the benign disease Treg groups, a total of 24 activated pathways were enriched (**Supplementary Table 5**). Most of the canonical secretome pathways were specific in E-MTAB-7961 kidney regeneration Treg, including Hepatic Fibrosis Signaling Pathway, PDGF Signaling, VEGF Signaling, and VEGF Family Ligand-Receptor Interactions, which functions were related to the tissue repair. IL-6 Signaling was specifically enriched in GSE50096 muscle injury 4D Treg, which was Treg in the muscle during the acute reaction phase after muscle injury.

**Table 2A:** B.

	Spleen	Spleen	Muscle	Muscle	Kidney	Kidney	
Canonical secretome pathway	GSE50096 4D	GSE50096 2W	GSE50096 4D	GSE50096 2W	E-MTAB-7961 F	E-MTAB-7961 R	Total
p38 MAPK Signaling	↑	↑	↑	↑	↑		5
STAT3 Pathway					↑	↑	2
Toll-like Receptor Signaling					↑	↑	2
IL-6 Signaling			↑				
Shared (Total)	1 (1)	1 (1)	1 (2)	1(1)	3 (3)	2 (22)	
Specific = total - shared	0	0	1	0	0	20	

20 Pathways in E-MTAB-7961 regeneration were listed in supplementary data (**Supplementary Table 2A**).

Table 2Bp38 MAPK Signaling was commonly shared in all the 3 Treg groups, indicating it was a conserved canonical secretome functions of Treg cells. A. Canonical secretome gene numbers in malignant disease Treg groups.

**Table:** 

Datasets	Category	Treg source	Type	TotalDEGs	CS up	/DEGs%	CS down	/DEGs%
GSE116347	Lymphoid	Tumor bearing mouse (B16) spleen	RNA-seq	1218	125	10.26	49	4.02
GSE116347	Lymphoid	Tumor bearing mouse (MC38) spleen	RNA-seq	1298	147	11.33	69	5.32
GSE116347	Lymphoid	Tumor bearing mouse (CT26) spleen	RNA-seq	905	103	11.38	38	4.20
GSE120280	Lymphoid	Tumor bearing mouse (TC-1) spleen	RNA-seq	1776	194	10.92	96	5.41
GSE139372	Non-lymphoid	Melanoma Skin	RNA-seq	1571	71	4.52	158	10.06
GSE120280	Non-lymphoid	Tumor bearing mouse (TC-1) tumor	RNA-seq	1933	161	8.33	97	5.02
GSE89225	Non-lymphoid	Breast Tumor	RNA-seq	1493	83	5.56	122	8.17
GSE116347	Non-lymphoid	Tumor bearing mouse (B16) tumor	RNA-seq	2462	173	7.03	138	5.61
GSE116347	Non-lymphoid	Tumor bearing mouse (MC38) tumor	RNA-seq	1651	143	8.66	90	5.45
GSE116347	Non-lymphoid	Tumor bearing mouse (CT26) tumor	RNA-seq	1393	61	4.38	182	13.07

CS, canonical secretome; DEG, differentially expressed gene. IPA analysis was performed to identify commonly shared upregulated canonical secretome pathways in malignant disease spleen Treg and tumor tissue Treg. A total of 17 activated canonical secretome pathways were enriched in malignant Treg groups. The most commonly shared pathways were p38 MAPK Signaling and IL-6 Signaling. Specifically enriched pathways in malignant spleen Treg and tumor tissue Treg were as following: HMGB1 Signaling and Cardiac Hypertrophy Signaling (Enhanced) were enriched in GSE116347 B16 spleen Treg. Type I Diabetes Mellitus Signaling was enriched in GSE116347 MC38 spleen Treg. GP6 Signaling Pathway and CDK5 Signaling were enriched in GSE120280 TC-1 spleen Treg. Pancreatic Adenocarcinoma Signaling, ILK Signaling and Antiproliferative Role of TOB in T Cell Signaling were enriched in GSE116347 MC38 tumor Treg. SPINK1 Pancreatic Cancer Pathway, PTEN Signaling, and Inhibition of Matrix Metalloproteases were enriched in GSE116347 CT26 tumor Treg. LPS/IL-1 Mediated Inhibition of RXR Function was enriched in GSE139372 melanoma Treg. Osteoarthritis Pathway was enriched in GSE89255 breast cancer Treg. (cut off: p < 0.05 and |Z-score| >= 2)

**Table 2B:** B

Canonical secrotome pathway	Spleen	Spleen	Spleen	Spleen	Mouse Melanoma Tumor	Mouse colon Tumor		Mouse lung Tumor	Melanoma	Breast cancer	
	GSE116347 B16	GSE116347 MC38	GSE116347 CT26	GSE120280 TC-1	GSE116347 B16	GSE116347 MC38	GSE116347 CT26	GSE120280 TC-1	GSE139372	GSE89255	Total
p38 MAPK Signaling	↑								↑	↑	3
IL-6 Signaling	↑	↑			↑						3
TREM1 Signaling		↑						↑			2
Toll-like Receptor Signaling		↑		↑							2
Shared (Total)	2(4)	3(4)	0	1(3)	1(1)	0(3)	0(3)	1(1)	1(2)	1(2)	
Specific = total - shared	2	1	0	2	0	3	3	0	1	1	

As shown in **Table 2B**, canonical secretomic genes were upregulated in Treg from tumor bearing spleen (B16, 125 genes), Treg from tumor bearing spleen (MC38, 147 genes), Treg from tumor bearing spleen (CT26, 103 genes), Treg from tumor bearing spleen (TC-1, 194 genes), Treg from melanoma skin (71 genes), Treg from TC-1 tumor (161 genes), Treg from breast tumor (83 genes), Treg from B16 tumor (173 genes), Treg from MC38 tumor (143 genes) and Treg from CT26 tumor (61 genes), respectively. Of note, the sources of Tregs were specified in the **Table 2B** and were also detailed in **Table 1**. A total of 17 activated canonical secretome pathways were enriched in Treg from malignant groups. The most common shared pathways were p38 MAPK signaling and IL-6 signaling. In addition, TREM1 signaling was shared in splenic Treg from MC38 tumor bearing mouse, and Treg from TC-1 lung tumor. Toll-like receptor signaling was shared by splenic Treg from mouse bearing MC38 tumor and splenic Treg from mouse bearing TC-1 lung tumor. Specifically enriched pathways in malignant spleen and tissues were as following: Two pathways such as HMGB1 signaling and cardiac hypertrophy signaling (Enhanced) were enriched in GSE116347 B16 splenic Treg. One pathway, type I diabetes mellitus signaling, was enriched in GSE116347 MC38 splenic Treg. Two pathways including GP6 signaling pathway and CDK5 signaling were enriched in GSE120280 TC-1 splenic Treg. Three pathways including pancreatic adenocarcinoma signaling, ILK signaling and antiproliferative role of TOB in T cell signaling were enriched in GSE116347 MC38 tumor Treg. Also, three pathways including SPINK1 pancreatic cancer pathway, PTEN signaling, and inhibition of matrix metalloproteases were enriched in GSE116347 CT26 tumor Treg. One pathway, LPS/IL-1 mediated inhibition of RXR function, was enriched in GSE139372 melanoma Treg. One pathway, osteoarthritis pathway, was enriched in GSE89255 breast cancer Treg. Taken together, these results have demonstrated that *1)* four Treg pathways including p38 MAPK signaling, IL-6 signaling, TREM1 signaling and Toll-like receptor signaling often found in non-malignant Treg are only shared in some tumor Treg but not all the tumor Treg groups (**Table 2B**); *2)* splenic Treg from mouse bearing different tumors upregulated different pathways; and *3)* tumor Treg upregulated specific pathways.

### Normal Tissue Tregs and Diseased Tissue Tregs Upregulate 56 Exosome Secretomic Pathways (ESP), Tumor Treg ESP Are Enriched With Vitamin-C Transport and Endothelin-1 Signaling, and Are More Focused Than Other Treg Groups

In the most comprehensive exosome database (http://www.exocarta.org/), we collected all the exosome proteins, 6560, which are experimentally identified in exosomes having no overlaps with other secretomes and no duplications. As shown in **Table 3A**, tissue Treg upregulated exosome secretomic genes including splenic Treg (9 genes), LN Treg (116 genes), splenic Treg (RNA-Seq, 301 genes), small intestine Treg (189 genes), BAT Treg (55 genes), VAT Treg (254 genes) and kidney Treg (356 genes), respectively. Of note, the sources of Tregs were specified in the **Table 3A** and were also detailed in **Table 1**. As shown in **Table 3A**, A total of 56 exosome pathways were enriched in normal group, including 22 shared and 34 specific pathways. Two pathways including synaptic long term depression and role of NFAT in regulation of the immune response were common in Treg from lymphoid tissues. Three pathways including cardiac hypertrophy signaling, sphingosine-1-phosphate signaling and Wnt/Ca+ pathway were common in Treg from non-lymphoid tissues. Production of nitric oxide and reactive oxygen species in macrophages was enriched in GSE37532 LN Treg. Six pathways including neuroinflammation signaling pathway, type I diabetes mellitus signaling, ERK5 signaling, NF-κB signaling, synaptic long term potentiation, and endothelin-1 signaling were enriched in E-MTAB-7961 splenic Treg. Three pathways including vitamin-C transport, FGF signaling, and senescence pathway were enriched in GSE37532 VAT Treg. 24 pathways enriched in E-MTAB-7961 kidney Treg were shown in supplementary **Table 3A**.

Table 3A70.6% (20/34) specific exosome pathways were in E-MTAB-7961 kidney tissue Treg. A list of human 6560 exosome genes were collected. A. Exosome gene numbers in normal Treg groups.

**Table:** 

Datasets	Category	Treg source	Type	TotalDEG	Exo up	/DEG%	Exo down	/DEG%
GSE119169	Lymphoid	Spleen	Array	44	9	20.45	5	11.36
GSE37532	Lymphoid	Lymph node	Array	397	116	29.22	24	6.05
E-MTAB-7961	Lymphoid	Spleen	RNA-seq	1802	301	16.70	107	5.94
GSE20366	Non-lymphoid	Small intestinal lamina propria	Array	756	189	25.00	165	21.83
GSE64909	Non-lymphoid	Brown adipose tissue	Array	113	55	48.67	5	4.42
GSE37532	Non-lymphoid	Visceral adipose tissue(VAT)	Array	924	254	27.49	85	9.20
E-MTAB-7961	Non-lymphoid	Kidney	RNA-seq	1584	356	22.47	174	10.98

The DEGs belong to the exosome genes in each Treg datasets were analyzed by IPA. Upregulated commonly shared exosome pathways in normal tissues were shown below. A total of 56 exosome pathways were enriched in normal Treg groups, including 22 commonly shared and 34 specific pathways. Synaptic Long Term Depression and Role of NFAT in Regulation of the Immune Response were common in lymphoid tissue Treg. Cardiac Hypertrophy Signaling, Sphingosine-1-phosphate Signaling and Wnt/Ca+ pathway were common in non-lymphoid tissue Treg. Production of Nitric Oxide and Reactive Oxygen Species in Macrophages was enriched in GSE37532 lymph node Treg. Neuroinflammation Signaling Pathway, Type I Diabetes Mellitus Signaling, ERK5 Signaling, NF-κB Signaling Synaptic Long Term Potentiation, and Endothelin-1 Signaling were enriched in E-MTAB-7961 spleen Treg. Vitamin-C Transport, FGF Signaling,and Senescence Pathway were enriched in GSE37532 VAT Treg. 24 pathways enriched in E-MTAB-7961 kidney were shown in **Supplementary Table 6A** (cut off: p < 0.05 and |Z-score| >= 2).

**Table 3A:** B.

Exosome Pathway	Spleen	Lymph Node	Spleen	SILP	BAT	VAT	Kidney	
	GSE119169	GSE37532	E-MTAB-7961	GSE20366	GSE64909	GSE37532	E-MTAB-7961	Total
Thrombin Signaling		↑	↑				↑	3
Signaling by Rho Family GTPases		↑	↑				↑	3
Role of NFAT in Cardiac Hypertrophy		↑	↑				↑	3
Phospholipase C Signaling		↑	↑				↑	3
p70S6K Signaling		↑	↑				↑	3
Estrogen Receptor Signaling			↑			↑	↑	3
Adrenomedullin signaling pathway			↑			↑	↑	3
IL-15 Production			↑			↑	↑	3
Synaptic Long Term Depression		↑	↑					2
Role of NFAT in Regulation of the Immune Response		↑	↑					2
Rac Signaling		↑					↑	2
Phospholipases			↑			↑		2
Cardiac Hypertrophy Signaling (Enhanced)			↑				↑	2
UVA-Induced MAPK Signaling			↑				↑	2
T Cell Exhaustion Signaling Pathway			↑				↑	2
Glioblastoma Multiforme Signaling			↑				↑	2
Chemokine Signaling			↑				↑	2
PI3K Signaling in B Lymphocytes			↑				↑	2
PDGF Signaling			↑				↑	2
Cardiac Hypertrophy Signaling						↑	↑	2
Sphingosine-1-phosphate Signaling						↑	↑	2
Wnt/Ca+ pathway						↑	↑	2
Shared (Total)	0	8(9)	18(24)	0	0	7(10)	19(43)	
Specific = total - shared	0	1	6	0	0	3	24	

SILP, Small intestinal lamina propria Treg; BAT, Brown adipose tissue Treg; VAT, Visceral adipose tissue Treg.

As shown in **Table 3B**, tissue Treg upregulated exosome secretomic genes including splenic Treg from injured skeletal muscle for 2 weeks (96 genes), splenic Treg from injured skeletal muscles for 4 days (115 genes), fibrosis kidney Treg (379 genes), regeneration kidney Treg (541 genes), Treg from injured skeletal muscle for 2 weeks (147 genes), and Treg from injured skeletal muscle for 4 days (94 genes), respectively. Of note, the sources of Tregs were specified in the **Table 3B** and were also detailed in **Table 1**.As shown in **Table 3B**, A total of 111 exosome pathways were enriched in benign disease groups including 32 shared pathways and 79 specific pathways. Among them, 71 out of 79 specifically enriched pathway were in Treg from regeneration kidney and fibrosis kidney (E-MTAN-7961 dataset). Vitamin-C transport and Th2 pathway were enriched in splenic Treg from injured skeletal muscle for 2 weeks (GSE50096). TREM1 signaling was enriched in splenic Treg from injured skeletal muscle for 4 days (GSE50096). PTEN signaling was enriched in injured skeletal muscle Treg for 4 days (GSE50096). Three pathways including dendritic cell maturation, apelin endothelial signaling pathway, and bladder cancer signaling were enriched in Treg from fibrosis kidney (E-MTAB-7961), and 71 specifically enriched pathway in Treg from regeneration kidney (E-MTAB-7961) were shown in supplementary **Table 3B**.

Table 3BE-MTAB-7961 Kidney regeneration Treg enriched pathways contributed to 91.0% (101/111) exosome pathways in identified benign disease Treg groups. A. Exosome gene numbers in benign disease Treg groups.

**Table:** 

Datasets	Category	Treg source	Type	TotalDEG	Exo up	/DEG%	Exo down	/DEG%
GSE50096	Lymphoid	Skeletal muscle injured 2w spleen	Array	308	96	31.17	28	9.09
GSE50096	Lymphoid	Skeletal muscle injured 4d spleen	Array	313	115	36.74	22	7.03
E-MTAB-7961	Non-lymphoid	Fibrosis kidney	RNA-seq	2440	379	15.53	109	4.47
E-MTAB-7961	Non-lymphoid	Regeneration kidney	RNA-seq	2365	541	22.88	131	5.54
GSE50096	Non-lymphoid	Skeletal muscle injured 2w muscle	Array	539	147	27.27	75	13.91
GSE50096	Non-lymphoid	Skeletal muscle injured 4d muscle	Array	465	94	20.22	101	21.72

IPA analysis was performed to identify upregulated commonly shared exosome pathways in benign disease tissue Treg. A total of 111 exosome pathways were enriched in benign disease Treg groups including 32 commonly shared and 79 specific pathways. The 71 of 79 specifically enriched pathways were in E-MTAN-7961 Treg. Vitamin-C Transport and Th2 Pathways were enriched in GSE50096 2W spleen Treg. TREM1 Signaling was enriched in GSE50096 4D spleen Treg. PTEN Signaling was enriched in GSE50096 4D muscle Treg. Dendritic Cell Maturation, Apelin Endothelial Signaling Pathway, and Bladder Cancer Signaling were enriched in E-MTAB-7961 fibrosis Treg. The 71 specifically enriched pathways in E-MTAB-7961 regeneration Treg were shown in **Supplementary Table 6B**. (cut off: p < 0.05 and |Z-score| >= 2).

**Table 3b:** B.

Exosome Pathway	Spleen	Spleen	Kidney		Muscle		
	GSE50096 2W	GSE50096 4D	E-MTAB-7961 F	E-MTAB-7961 R	GSE50096 2W	GSE50096 4D	Total
Cardiac Hypertrophy Signaling	↑	↑	↑	↑			4
Role of NFAT in Regulation of the Immune Response	↑	↑	↑				3
Synaptic Long Term Depression		↑	↑	↑			3
PI3K Signaling in B Lymphocytes		↑	↑	↑			3
Adrenomedullin signaling pathway		↑	↑	↑			3
Chemokine Signaling		↑	↑	↑			3
Xenobiotic Metabolism CAR Signaling Pathway	↑					↑	2
Endocannabinoid Neuronal Synapse Pathway		↑	↑				2
Necroptosis Signaling Pathway		↑		↑			2
Neuroinflammation Signaling Pathway		↑		↑			2
Reelin Signaling in Neurons			↑	↑			2
White Adipose Tissue Browning Pathway			↑	↑			2
Hepatic Fibrosis Signaling Pathway			↑	↑			2
GDNF Family Ligand-Receptor Interactions			↑	↑			2
UVA-Induced MAPK Signaling			↑	↑			2
Glioblastoma Multiforme Signaling			↑	↑			2
STAT3 Pathway			↑	↑			2
IL-8 Signaling			↑	↑			2
Phospholipases			↑	↑			2
Sphingosine-1-phosphate Signaling			↑	↑			2
Wnt/Ca+ pathway			↑	↑			2
Cholecystokinin/Gastrin-mediated Signaling			↑	↑			2
PDGF Signaling			↑	↑			2
p70S6K Signaling			↑	↑			2
IL-15 Production			↑	↑			2
Estrogen-Dependent Breast Cancer Signaling			↑	↑			2
Estrogen Receptor Signaling			↑	↑			2
Factors Promoting Cardiogenesis in Vertebrates			↑	↑			2
Thrombin Signaling			↑	↑			2
FcγRIIB Signaling in B Lymphocytes			↑	↑			2
Endothelin-1 Signaling			↑	↑			2
Glutathione-mediated Detoxification			↑	↑			2
T Cell Exhaustion Signaling Pathway				↑	↑		2
Shared (Total)	3(5)	9(10)	29(32)	30(101)	1(1)	1(2)	
Specific = total – shared	2	1	3	71	0	1	

As shown in **Table 3C**, exosome secretomic genes were upregulated in splenic Treg from B16 tumor bearing mouse (328 genes), splenic Treg from MC38 tumor bearing mouse (331 genes), splenic Treg from CT26 tumor bearing mouse (236 genes), splenic Treg from TC-1 tumor bearing mouse (475 genes), Treg from melanoma skin (121 genes), Treg from TC-1 tumor (419 genes), Treg from breast tumor (204 genes), Treg from B16 tumor (572 genes), Treg from MC38 tumor (415 genes) and Treg from CT26 tumor (188 genes), respectively. Of note, the sources of Tregs were specified in the **Table 3C** and were also detailed in **Table 1**. As shown in **Table 3C**, a total of 69 exosome pathways were enriched in malignant groups, including 35 common shared and 34 specific pathways. The 42 out of 69 exosome pathways were in the lymphoid tissues. Two pathways including vitamin-C transport and endothelin-1 signaling were the most commonly enriched pathways. Eight pathways including cell cycle control of chromosomal replication, BEX2 signaling pathway, cholesterol biosynthesis III (via desmosterol), superpathway of cholesterol biosynthesis, cholesterol biosynthesis II (via 24,25-dihydrolanosterol), cholesterol biosynthesis I, and glycolysis I were only common shared in Treg from malignant non-lymphoid tissues.

**Table 3C:** Lymphoid tissue Treg enriched more exosome pathways than non-lymphoid tissue Treg. A. Exosome gene numbers in benign disease Treg groups.

Datasets	Category	Treg source	Type	TotalDEG	Exo up	/DEG%	Exo down	/DEG%
GSE116347	Lymphoid	Tumor bearing mouse (B16) spleen	RNA-seq	1218	328	26.93	113	9.28
GSE116347	Lymphoid	Tumor bearing mouse (MC38) spleen	RNA-seq	1298	331	25.50	147	11.33
GSE116347	Lymphoid	Tumor bearing mouse (CT26) spleen	RNA-seq	905	236	26.08	92	10.17
GSE120280	Lymphoid	Tumor bearing mouse (TC-1) spleen	RNA-seq	1776	475	26.75	158	8.90
GSE139372	Non-lymphoid	Melanoma Skin	RNA-seq	1571	121	7.70	287	18.27
GSE120280	Non-lymphoid	Tumor bearing mouse (TC-1) tumor	RNA-seq	1933	419	21.68	270	13.97
GSE89225	Non-lymphoid	Breast Tumor	RNA-seq	1493	204	13.66	250	16.74
GSE116347	Non-lymphoid	Tumor bearing mouse (B16) tumor	RNA-seq	2462	572	23.23	375	15.23
GSE116347	Non-lymphoid	Tumor bearing mouse (MC38) tumor	RNA-seq	1651	415	25.14	268	16.23
GSE116347	Non-lymphoid	Tumor bearing mouse (CT26) tumor	RNA-seq	1393	188	13.50	376	26.99

IPA analysis was performed to identify upregulated commonly shared exosome pathways in malignant disease tissue Treg (cut off: p < 0.05 and |Z-score| >= 2). A total of 69 exosome pathways enriched in malignant Treg groups, including 35 commonly shared pathways and 34 specific pathways. The 42 of 69 exosome pathways were identified in the lymphoid tissue Treg. Vitamin-C Transport and Endothelin-1 Signaling were the most common enriched pathways. Cell Cycle Control of Chromosomal Replication, Cell Cycle Control of Chromosomal Replication, BEX2 Signaling Pathway, Cholesterol Biosynthesis III (via Desmosterol), Superpathway of Cholesterol Biosynthesis, Cholesterol Biosynthesis II (via 24,25-dihydrolanosterol), Cholesterol Biosynthesis I and Glycolysis I were only commonly shared in non-lymphoid tissue Treg. Exo, exosome; DEG, differentially expressed gene; DEG%, percentage in total DEG numbers.

**Table 3C:** B.

Exosome Pathway	Spleen		Spleen		Spleen		Melanoma	Mouse lung Tumor	Breast cancer	Mouse Melanoma Tumor	Mouse colon Tumor		
	GSE116347 B16	GSE116347 MC38		GSE116347 CT26		GSE120280 TC-1	GSE139372	GSE120280 TC-1	GSE89255	GSE116347 B16	GSE116347 MC38	GSE116347 CT26	Total
Vitamin-C Transport	↑	↑		↑		↑				↑	↑		6
Role of NFAT in Regulation of the Immune Response	↑	↑		↑		↑							4
Endothelin-1 Signaling	↑	↑		↑		↑		↑			↑		6
Endocannabinoid Neuronal Synapse Pathway	↑	↑		↑		↑							4
Glioblastoma Multiforme Signaling	↑	↑		↑									3
TREM1 Signaling	↑	↑		↑									3
Thrombin Signaling	↑	↑		↑				↑			↑		5
Wnt/Ca+ pathway	↑	↑		↑									3
Dendritic Cell Maturation	↑	↑				↑							3
Glutathione-mediated Detoxification	↑	↑				↑							3
Signaling by Rho Family GTPases	↑			↑		↑							3
PI3K Signaling in B Lymphocytes	↑			↑		↑		↑			↑		5
Synaptic Long Term Depression	↑			↑									2
Estrogen Receptor Signaling	↑			↑									2
UVA-Induced MAPK Signaling	↑			↑				↑			↑		4
Chemokine Signaling	↑			↑									2
Phospholipases	↑			↑									2
Death Receptor Signaling	↑			↑									2
Cardiac Hypertrophy Signaling	↑					↑							2
Phospholipase C Signaling	↑					↑							2
IL-15 Production	↑					↑							2
Neuroinflammation Signaling Pathway		↑		↑									2
IL-8 Signaling				↑		↑		↑			↑		4
Rac Signaling				↑		↑		↑		↑			4
Cell Cycle Control of Chromosomal Replication								↑	↑	↑	↑		4
BEX2 Signaling Pathway									↑		↑		2
Cholesterol Biosynthesis III (via Desmosterol)										↑	↑		2
Superpathway of Cholesterol Biosynthesis										↑	↑		2
Cholesterol Biosynthesis II (via 24,25-dihydrolanosterol)										↑	↑		2
Cholesterol Biosynthesis I										↑	↑		2
Glycolysis I										↑	↑		2
Shared (Total)	21 (29)	11 (14)		19 (25)		13 (14)	0	7 (21)	2 (3)	8 (13)	13 (25)	0	
Specific = total - shared	8	3		6		1	0	14	1	5	12	0	

All specific pathways will put in supplementary data (**Supplementary Table 6C**).

Taken together, our results have demonstrated that *First*, in comparison to that shown in **Tables 3A**, **B**, Treg from tumor bearing spleens and tumor tissues in **Table 3C** upregulate more exosome secretomic genes but less pathways, suggesting that Treg pathways from tumors are more focused than Treg from non-malignant disease tissues; *Second*, two pathways including synaptic long term depression and role of NFAT in regulation of the immune response were common in normal lymphoid tissue; three pathways including cardiac hypertrophy signaling, sphingosine-1-phosphate signaling and Wnt/Ca+ pathway were common in Treg from non-lymphoid tissues; *Third*, Treg from non-malignant disease groups have much less shared pathways than normal tissue Treg and malignant tissue Treg; and *F*o*urth*, vitamin-C transport and endothelin-1 signaling were most common enriched pathways; and eight pathways were enriched in Treg from malignant non-lymphoid tissues in cholesterol synthesis and cell cycle controls.

### Normal Tissue Tregs, Diseased Tissue Tregs and Tumor Tregs Upregulate Novel Innate Immune Caspase-1 Secretomic Genes and Innate Immune Caspase-4 Secretomic Genes to Fulfill Their Tissue-Specific, Secretomes-Specific Functions and Shared Functions

It has been reported that NLRP3 negatively regulate Treg differentiation through Kpna-2 mediated nuclear translocation (120); that TLR2 and TLR4 signalings induce inflammasome priming in Th1-like Treg (128); and that caspase-1 canonical inflammasome pathway and caspase-4 (humans)/caspase-11 (mice) non-canonical inflammasome pathway all cleave N-terminal Gasdermin D (123), which form a protein pore/channel on the cell membrane to release proinflammatory cytokines such as IL-1β and IL-18. It has been also reported that caspase-1-dependent non-canonical secretome (non-signal peptide-mediated) secrete 961 proteins (102), and caspase-4-dependent non-canonical secretome secrete 1,223 proteins (103). We hypothesized that caspase-1- dependent secretomic transcripts and caspase-4-dependent secretomic transcripts are modulated in Treg from normal tissues, non-malignant disease Treg, and Treg from tumor spleens and tumors. As shown in **Table 3D**, Treg from normal LN and spleen upregulated 8 to15 caspase-1-dependent secretomic genes and 12 to 35 caspase-4 secretomic genes, respectively. In addition, intestine Treg, BAT Treg, VAT Treg and kidney Treg upregulated 16, 8, 18 and 20 casapse-1 secretomic genes, and 15, 8, 22, and 42 casapse-4 secretomic genes, respectively. Treg from injured skeletal muscle (2 weeks) spleen and Treg from injured skeletal muscle (4 days) spleen, fibrosis kidney Treg, regeneration kidney Treg, injured skeletal muscle (2 weeks) Treg, and injured skeletal muscle (4 days) Treg upregulated 6, 7, 13, 25, 10, and 10 caspase-1 secretomic genes, and upregulated 10, 13, 37, 56, 11, and 10 caspase-4 secretomic genes, respectively. Moreover, Treg from B16 spleen, Treg from MC38 spleen, Treg from CT26 spleen, Treg from TC-1 spleen upregulated 20, 18, 14, 21 caspase-1 secretomic genes, and 35, 42, 28, and 38 caspase-4 secretomic genes, respectively. Furthermore, Treg from melanoma, Treg from TC-1 tumor, Treg from breast tumor, Treg from B16 tumor, Treg from MC38 tumor, and Treg from CT26 tumor upregulated 2, 18, 9, 42, 27, and 9 caspase-1 secretomic genes, and 8, 33, 16, 72, 39, and 11 caspase-4 secretomic genes, respectively. Of note, the sources of Tregs were specified in the **Table 3D** and were also detailed in **Table 1**.

**Table 3D:** Gene numbers of caspase-4 dependent GSDMD secretomes were more than caspase-1 dependent GSDMD secretomes in various Treg groups.

Datasets	Category	Treg source	Type	Total DEG	Casp-1up	/DEG%	Casp-1down	/DEG%	Casp-4 up	/DEG%	Casp-4 down	/DEG%
GSE119169	Lymphoid	Spleen	Array	44	0	0.00	0	0.00	2	4.55	0	0.00
GSE37532	Lymphoid	Lymph node	Array	397	8	2.02	0	0.00	12	3.02	0	0.00
E-MTAB-7961	Lymphoid	Spleen	RNA-seq	1802	15	0.83	3	0.17	35	1.94	9	0.50
GSE20366	Non-lymphoid	Small intestinal lamina propria	Array	756	16	2.12	11	1.46	15	1.98	11	1.46
GSE64909	Non-lymphoid	Brown adipose tissue	Array	113	8	7.08	0	0.00	8	7.08	0	0.00
GSE37532	Non-lymphoid	Visceral adipose tissue(VAT)	Array	924	18	1.95	2	0.22	22	2.38	5	0.54
E-MTAB-7961	Non-lymphoid	Kidney	RNA-seq	1584	20	1.26	4	0.25	42	2.65	5	0.32
GSE50096	Lymphoid	Skeletal muscle injured 2w spleen	Array	308	6	1.95	0	0.00	10	3.25	1	0.32
GSE50096	Lymphoid	Skeletal muscle injured 4d spleen	Array	313	7	2.24	0	0.00	13	4.15	1	0.32
E-MTAB-7961	Non-lymphoid	Fibrosis kidney	RNA-seq	2440	13	0.83	4	0.25	37	2.36	8	0.51
E-MTAB-7961	Non-lymphoid	Regeneration kidney	RNA-seq	2365	25	1.06	6	0.25	56	2.37	7	0.30
GSE50096	Non-lymphoid	Skeletal muscle injured 2w muscle	Array	539	10	1.86	0	0.00	11	2.04	5	0.93
GSE50096	Non-lymphoid	Skeletal muscle injured 4d muscle	Array	465	10	2.15	2	0.43	10	2.15	8	1.72
GSE116347	Lymphoid	Tumor bearing mouse (B16) spleen	RNA-seq	1218	20	1.64	3	0.25	35	2.87	3	0.25
GSE116347	Lymphoid	Tumor bearing mouse (MC38) spleen	RNA-seq	1298	18	1.39	2	0.15	42	3.24	4	0.31
GSE116347	Lymphoid	Tumor bearing mouse (CT26) spleen	RNA-seq	905	14	1.55	1	0.11	28	3.09	2	0.22
GSE120280	Lymphoid	Tumor bearing mouse (TC-1) spleen	RNA-seq	1776	21	1.18	9	0.51	38	2.14	11	0.62
GSE139372	Non-lymphoid	Melanoma Skin	RNA-seq	1571	2	0.13	8	0.51	8	0.51	19	1.21
GSE120280	Non-lymphoid	Tumor bearing mouse (TC-1) tumor	RNA-seq	1933	18	0.93	6	0.31	33	1.71	13	0.67
GSE89225	Non-lymphoid	Breast Tumor	RNA-seq	1493	9	0.60	6	0.40	16	1.07	18	1.21
GSE116347	Non-lymphoid	Tumor bearing mouse (B16) tumor	RNA-seq	2462	42	1.71	9	0.37	72	2.92	23	0.93
GSE116347	Non-lymphoid	Tumor bearing mouse (MC38) tumor	RNA-seq	1651	27	1.64	11	0.67	39	2.36	21	1.27
GSE116347	Non-lymphoid	Tumor bearing mouse (CT26) tumor	RNA-seq	1393	9	0.65	16	1.15	11	0.79	52	3.73

GSDMD, Gasdermin-D; Casp-1, caspase-1; Casp-4, caspase-4; DEG, differentially expressed gene. However, the total numbers in each Treg datasets was too low to perform IPA analysis. The detailed lists were shown in **Supplementary Table 7**.

**Figure 5:**
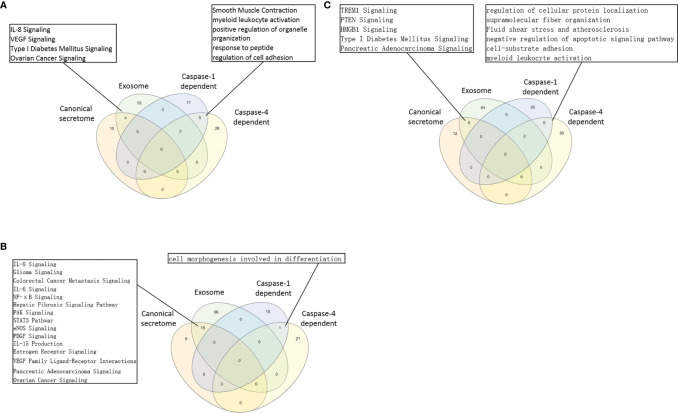
**(A)** In normal tissue Treg groups, 4 pathways were shared by Treg canonical secretomes and exosome secretomes. The 5 pathways in Treg groups were shared by caspase-1-dependent, and Caspase-4 dependent gasdermine-D secretomes. Canonical secretome, Exosome secretomes, Caspase-1-dependent and Caspase-4 dependent gasdermine-D pathways in normal spleen Treg and tissue Treg were analyzed by Venn diagram. The Treg canonical secretomes and Treg exosomes had 4 overlapped pathways. The caspase-1 dependent-gasdermin D secretomes and Caspase-4 dependent gasdermine-D secretomes had 5 overlapped pathways. **(B)** In benign disease tissue Treg groups, 15 pathways were shared by Treg canonical secretomes and Treg Exosome secretomes. Only one pathway was shared by Treg caspase-1-gasdermin D secretomes and Treg caspase-4 dependent gasdermine-D secretomes. **(C)** In malignant disease tissue Treg groups, 5 pathways were shared by Treg canonical secretomes and Treg Exosome secretomes. The 6 pathway was shared by Treg caspase-1-gasdermin D secretomes and Treg caspase-4 dependent gasdermine-D secretomes.

The pathway analysis results (**Table 3E** and **Figure 5A**) showed that normal tissue Treg and non-tumor diseased tissue Treg shared seven upregulated caspase-1 secretomic gene pathways including pathways in cancer, vesicle organization, negative regulation of apoptotic signaling, myeloid leukocyte activation, striated muscle cell differentiation, cell morphogenesis, and supramolecular fiber organization. Treg from non-tumor diseased tissues and tumors shared three upregulated caspase-1 secretomic pathways including extracellular structure, response to reactive oxygen species, cell-substrate junction. Tumor spleen Treg and tumor Treg shared one upregulated caspase-1 secretomic pathway, fluid shear stress and atherosclerosis. In addition, normal spleen Treg, normal tissue Treg, non-tumor diseased spleen Treg, non-tumor diseased tissue Treg, tumor spleen Treg and tumor Treg had 2, 13, 9, 11, and 12 specific upregulated caspase-1 secretomic pathways, respectively.

**Table 3E:** Metascape analysis was performed to identify pathways associated with upregulated caspase-1 dependent GSDMD secretome gene lists in various Treg groups.

Enrichment	normal spleen	normal tissue	disease spleen	disease tissue	tumor spleen	tumor tissue
Pathways in cancer	+	+	+	+	+	+
vesicle organization	+		+		+	+
negative regulation of apoptotic signaling pathway		+			+	+
myeloid leukocyte activation	+				+	
striated muscle cell differentiation		+		+		
cell morphogenesis involved in differentiation		+		+		
supramolecular fiber organization		+				+
extracellular structure organization			+			+
response to reactive oxygen species				+		+
cell-substrate junction assembly				+		+
Fluid shear stress and atherosclerosis					+	+
positive regulation of organelle organization	+					
ECM-receptor interaction	+					
NABA MATRISOME ASSOCIATED		+				
morphogenesis of an epithelium		+				
extracellular matrix disassembly		+				
TNF signaling pathway		+				
establishment of organelle localization		+				
wound healing		+				
Smooth Muscle Contraction		+				
regulation of lymphocyte differentiation		+				
response to peptide		+				
angiogenesis		+				
regulation of cell adhesion		+				
Hypertrophic cardiomyopathy (HCM)		+				
Diseases of signal transduction by growth factor receptors and second messengers		+				
actomyosin structure organization				+		
Extracellular matrix organization				+		
organophosphate biosynthetic process				+		
branching morphogenesis of an epithelial tube				+		
epithelial cell development				+		
Hemostasis				+		
cellular response to external stimulus				+		
carbohydrate metabolic process				+		
Non-integrin membrane-ECM interactions				+		
response to oxidative stress					+	
post-translational protein modification					+	
coenzyme metabolic process					+	
cell-substrate adhesion					+	
cellular response to oxygen levels					+	
generation of precursor metabolites and energy					+	
positive regulation of cell motility					+	
MAPK signaling pathway					+	
Degradation of the extracellular matrix					+	
Proteoglycans in cancer					+	
regulation of body fluid levels					+	
regulation of cellular protein localization						+
positive regulation of binding						+
PID INTEGRIN4 PATHWAY						+
Post-translational protein phosphorylation						+
cell division						+
positive regulation of response to external stimulus						+
positive regulation of cell migration						+
chromatin remodeling						+
regulation of cholesterol metabolic process						+
response to hypoxia						+
purine nucleoside triphosphate metabolic process						+
Cell Cycle, Mitotic						+

The pathway analysis results (**Table 3F** and **Figure 5B**) showed that normal tissue Treg and non-tumor diseased tissue Treg shared 15 upregulated caspase-4 secretomic pathways. Treg from non-tumor diseased tissues and tumors shared four upregulated caspase-4 secretomic pathways. Tumor spleen Treg and tumor Treg shared one upregulated caspase-4 secretomic pathway, fluid shear stress and atherosclerosis. In addition, normal spleen Treg, normal tissue Treg, non-tumor diseased spleen Treg, non-tumor diseased tissue Treg, tumor spleen Treg and tumor Treg had 8, 10, 2, 9, 11, and 9 specific upregulated caspase-4 secretomic pathways, respectively.

**Table 3F:** Metascape analysis was performed to identify pathways associated with upregulated caspase-4 dependent GSDMD secretome gene lists in various Treg groups.

Enrichment	normal spleen	normal tissue	disease spleen	disease tissue	tumor spleen	tumor tissue
Signaling by Interleukins	+	+	+	+	+	+
regulation of peptidase activity	+	+			+	+
positive regulation of organelle organization	+	+			+	+
regulation of cell adhesion	+		+	+		
Fluid shear stress and atherosclerosis		+		+		+
PID AMB2 NEUTROPHILS PATHWAY		+		+		+
regulated exocytosis	+			+		
neuron death	+				+	
negative regulation of peptidase activity	+				+	
carbohydrate metabolic process	+				+	
response to oxygen levels		+		+		
blood vessel morphogenesis		+		+		
Tuberculosis		+			+	
response to wounding		+				+
myeloid leukocyte activation		+				+
positive regulation of cellular component movement				+	+	
response to carbohydrate				+		+
supramolecular fiber organization				+		+
response to extracellular stimulus				+		+
negative regulation of apoptotic signaling pathway					+	+
regulation of calcium-mediated signaling	+					
kaposi sarcoma-associated herpesvirus infection	+					
regulation of protein stability	+					
negative regulation of leukocyte activation	+					
extrinsic apoptotic signaling pathway	+					
antigen processing and presentation	+					
glucose homeostasis	+					
regulation of cytokine-mediated signaling pathway	+					
negative regulation of protein phosphorylation		+				
neuroinflammatory response		+				
Smooth Muscle Contraction		+				
PID CXCR4 PATHWAY		+				
prostanoid metabolic process		+				
extracellular structure organization		+				
positive regulation of response to external stimulus		+				
positive regulation of cell migration		+				
response to peptide		+				
positive regulation of protein binding		+				
leukocyte differentiation			+			
negative regulation of response to external stimulus			+			
regulation of cytoskeleton organization				+		
extracellular matrix disassembly				+		
aging				+		
regulation of small GTPase mediated signal transduction				+		
cell morphogenesis involved in differentiation				+		
regulation of system process				+		
regulation of MAPK cascade				+		
negative regulation of intracellular signal transduction				+		
small molecule catabolic process				+		
PID CASPASE PATHWAY					+	
Legionellosis					+	
cell activation involved in immune response					+	
Epstein-Barr virus infection					+	
response to bacterium					+	
positive regulation of epithelial cell migration					+	
African trypanosomiasis					+	
cell-substrate adhesion					+	
vascular endothelial growth factor receptor signaling pathway					+	
positive regulation of receptor binding					+	
regulation of I-kappaB kinase/NF-kappaB signaling					+	
regulation of cellular protein localization						+
regulation of protein binding						+
Pentose phosphate pathway						+
extracellular matrix organization						+
galactose catabolic process						+
monosaccharide catabolic process						+
regulation of small molecule metabolic process						+
Phagosome						+
response to toxic substance						+

Taken together, our results have demonstrated that *First*, upregulations of caspase-1- dependent secretomic genes and caspase-4-dependent secretomic genes in Treg are more than downregulation of those secretomic genes; *Second*, upregulations of caspase-4-dependent secretomic genes in Treg often are more than upregulations of caspase-1 secretomic genes in Treg; *Third*, most of Treg caspase-1 secretomic gene pathways are different from that of Treg caspase-4 secretomic pathways (**Figure 5C**), suggesting that Treg caspase-1 secretomic genes play different roles in Treg from that of caspase-4 secretomes; and *Fourth*, the majority of caspase-1 secretomic pathways and caspase-4 secretomic pathways are tissue Treg-specific. These results suggest that normal tissue Treg, non-tumor diseased tissue Treg and tumor Treg upregulate novel caspase-1 secretomic genes and caspase-4 secretomic genes to fulfill their tissue-specific, secretomes-specific Treg functions and shared Treg functions.

### Most Tissue Treg Transcriptomes Are Controlled by Foxp3; Tumor Tregs Had Significantly Increased Foxp3 Non-Collaboration Genes With ROS and 17 Other Pathways

**Figure 6:**
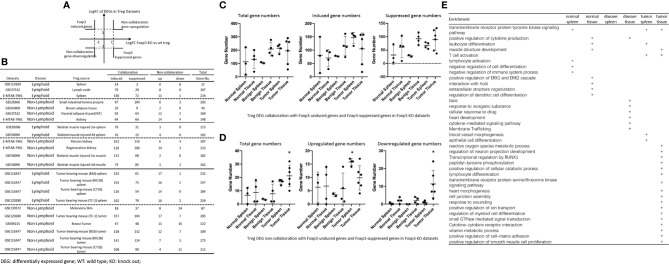
**(A, B)** All the upregulated and downregulated genes from Treg datasets were compared to Foxp3 deletion (Foxp3 KO) microarray dataset versus WT Treg dataset (GSE6681) to identify Foxp3-collaboration genes. The DEGs overlapped in the upregulated genes in Treg datasets and downregulated genes in Foxp3 KO dataset were defined as FOXP3-induced gene. The DEGs overlapped in the downregulated genes in Treg datasets and upregulated genes in Foxp3 KO datasets were defined as FOXP3-suppressed genes. Both groups of DEGs were defined as FOXP3 collaboration genes. Otherwise, the DEGs up-, or down-regulated genes that were not overlapped between Treg datasets and Foxp3 KO datasets were defined as non-collaboration genes. **(C, D)** In the DEGs shared between Treg datasets and Foxp3-regulated genes in Foxp3 KO datasets, the majority of which were FOXP3 collaborated genes, indicating Treg-master transcription factor FOXP3. The total non-collaboration DEG numbers were much higher in tumor tissue Treg groups (p < 0.05). The DEGs regulated in tumor tissue Treg groups were more complicated than that in benign and normal tissue Treg groups. **(E)** In FOXP3 non-collaboration gene pathways, normal tissue Treg groups shared 1 pathway with benign disease tissue Treg groups, and shared 3 pathways with tumor tissue Treg groups. FOXP3 non-collaboration genes in each Treg group were analyzed using the Metascape pathway analysis. The FOXP3 non-collaboration pathways in normal tissue Treg, benign tissue Treg, and malignant disease spleen and tissue Treg groups were summarized in this table. Venn diagram analysis was performed to show the overlapped pathways among the normal tissue Treg, benign diseased tissue Treg, and malignant tissue Treg groups. *Significantly changed.

It has been reported that Foxp3 plays significant roles in Treg specific gene expressions (74), and induces 58 gene upregulation and 46 gene downregulation (129). We hypothesized that Foxp3 plays significant roles in promoting DEGs in Treg from normal tissues, non-tumor diseased tissues and tumor tissues. To test this hypothesis, we placed all the DEGs of Treg versus Teffector cells with Treg upregulated genes in the >1 log2 in the Y-axis, and Foxp3 depletion-suppressed genes in the >1 log2 in the X-axis (**Figure 6A**). The argument was that if gene expressions are promoted by Foxp3, the genes will be showed in Foxp3 induced gene groups; otherwise, they will be showed in Foxp3 suppressed gene group. If any genes upregulated or downregulated in tissue Treg, non-tumor diseased Treg and tumor Treg are not collaborated with Foxp3 regulation, they will be placed in non-collaboration gene groups. As shown in **Figure 6B**, LN Treg, splenic Treg, intestine Treg, BAT Treg, VAT Treg, kidney Treg had 8, 11, 0, 2, 11, and 14 upregulated non-collaboration genes. In addition, Treg from injured skeletal muscle (2 weeks) spleen, Treg from injured skeletal muscle (4 days) spleen, Treg from fibrosis kidney, Treg from regeneration kidney, Treg from injured skeletal muscle (2 weeks), Treg from injured skeletal muscle (4 days) had 3, 4, 6, 14, 2, and 1 upregulated non-collaboration genes. Treg from B16 spleen, Treg from MC38 spleen, Treg from CT26 spleen, Treg from TC-1 spleen, Treg from melanoma, Treg from TC-1 tumor, Treg from breast tumor, Treg from B16 tumor, Treg from MC38 tumor, and Treg from CT26 tumor had 17, 16, 14, 16, 8, 17, 11, 12, 7 and 4 upregulated non-collaboration genes. Moreover, Treg from normal tissues had 0% to 12.2% non-collaboration genes, Treg from non-tumor diseased tissues had 0.6% to 7.3% non-collaboration genes, and Treg from tumor spleens and tumors had 3.6% to 36.8% non-collaboration genes (**Figures 6C, D**). Furthermore, as shown in the dot plots, Treg from tumor spleens and tumors had significantly increased Foxp3 non-collaboration genes. As shown in **Figure 6E**, 37 pathways were associated with Foxp3 non-collaboration genes, among which 18 pathways were specifically associated with tumor Treg. Our results have demonstrated that normal tissue Treg and non-tumor diseased tissue Treg have transcriptomic changes in Foxp3-dependent manners; and tumor spleen Treg and tumor Treg have certain transcriptomic changes in Foxp3 non-collaboration manners with ROS and other 17 specific pathways.

### PD-1 Does but CTLA-4 Does Not, Play Significant Roles in Promoting Treg Upregulated Genes in Normal Tissue and Non-Tumor Diseased Tissue Tregs; and Tumor Splenic and Tumor Tregs Have Certain CTLA-4-, and PD-1-, Non-Collaboration Transcriptomic Changes With Innate Immune Dominant Pathways

**Figure 7:**
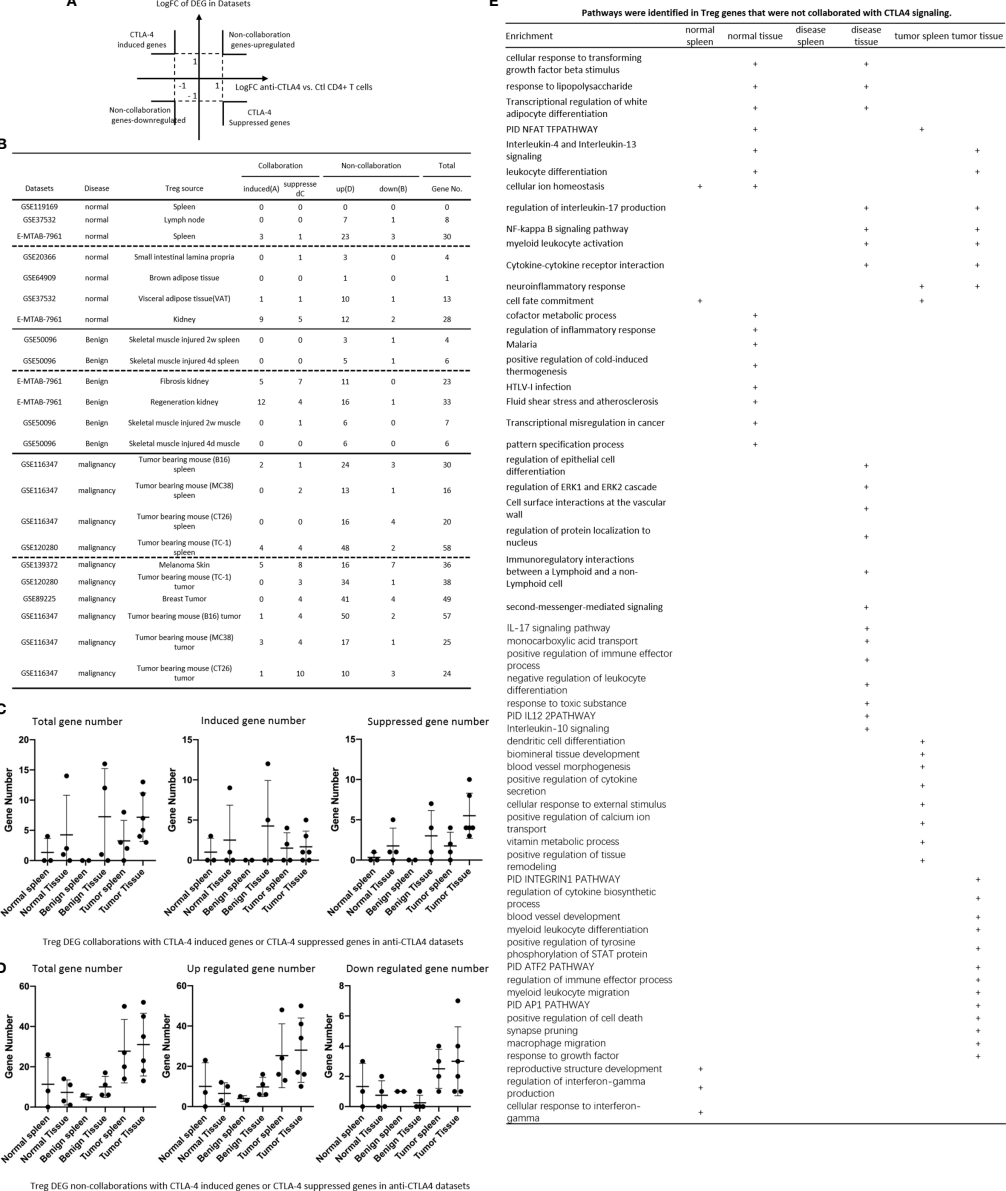
**(A, B)** All the upregulated genes and downregulated genes in tissue Treg groups were compared to anti-cytotoxic T cell antigen-4 (CTLA-4) antibody-treated Treg (CTLA-4 signaling suppressed) versus CTLA-4 signaling non-suppressed CONTROL CD4+ T cell dataset (GSE14716) to determine how many Treg genes were dependent on CTLA-4 signaling. **(C)** Treg DEG collaboration with CTLA-4 induced genes and CTLA-4 suppressed genes in anti-CTLA-4 datasets. **(D)** Treg DEG non-collaboration with CTLA-4 induced genes and CTLA-4 suppressed genes in anti-CTLA-4 datasets. **(E)** pathways identified in Treg genes and not collaborated with CTLA-4 signalling.

It has been reported that a Treg-specific deficiency of cytotoxic T lymphocyte antigen 4 (CTLA-4) results in development of systemic lymphoproliferation, fatal autoimmune disease, hyperproduction of immunoglobulin E, and potent tumor immunity (130). Our previous report determined the expression of 28 co-signaling receptors in 32 human tissues in physiological/pathological conditions and found that the expression of inflammasome components are correlated with the expression of co-signaling receptors (19). We also reported that among 10 immune checkpoint receptors (ICRs), lung, liver, spleen and intestine macrophages (Mφ) and bone marrow (BM) Mφ express higher levels of CD274 (programmed cell death-1, PDL-1) than adipose tissue Mφ, presumably to counteract the M1 Mφ dominant status *via* its reverse signaling behavior (1), suggesting that tissue immune checkpoint receptor signaling may also modulate Tregs. We hypothesized that CTLA-4 play roles in modulating DEGs in Treg from normal tissues, non-tumor diseased tissues and tumor tissues. To test this hypothesis, we placed all the CLTA-4-induced Treg genes cells with Treg upregulated genes in the >1 log2 in the Y-axis, and anti-CTLA-4 antibody treated (CTLA-4 suppressed) genes in the >1 log2 in the X-axis (**Figure 7**). The argument was that if gene expressions are promoted by CTLA-4, the genes will be shown in CTLA-4 induced gene groups; otherwise, they will be shown in CTLA-4 suppressed gene group. If any genes upregulated or downregulated in normal tissue Treg, non-tumor diseased Treg and tumor Treg are not collaborated with CTLA-4 regulation, they will be placed in non-collaboration gene groups. As shown in **Figure 7**, we found that there were a few non CTLA-4 collaboration genes upregulated in normal tissue and non-tumor diseased tissue Treg in spleen (23 genes), VAT Treg (10 genes) and kidney Treg (11-16 genes). In contrast, tumor Treg had non CTLA-4 collaboration genes upregulated ranging from 10 genes to 50 genes, which were higher than that of normal tissue Treg and non-tumor diseased tissue Treg. In addition, we found, as shown in **Figure 7E**, that 13 pathways associated with CTLA-4 non-collaboration genes in Treg shared in various groups; 8 pathways associated with CTLA-4 non-collaboration genes in normal tissue Treg; 13 pathways associated with Treg from non-tumor diseased tissues, and 21 pathways associated with tumor Treg.

**Figure 8:**
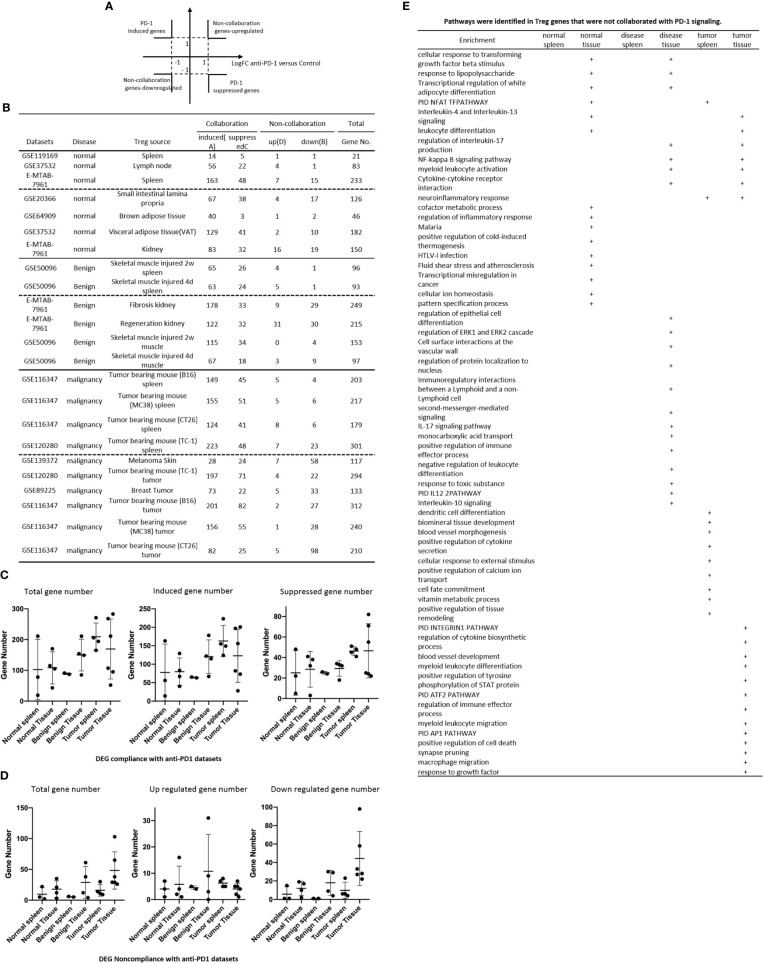
**(A, B)** All the upregulated genes and downregulated genes in tissue Treg groups were compared to anti-programmed death-1 (PD-1) antibody-treated Treg (PD-1 signaling suppressed) versus PD-1 signaling non-suppressed CONTROL CD4+ T cell dataset (GSE114300) to determine how many Treg genes were dependent on PD-1 signaling. **(C)** Treg DEG collaboration with PD-1 induced genes and PD-1 suppressed genes in anti-PD-1 datasets. **(D)** Treg DEG non-collaboration with PD-1 induced genes and PD-1 suppressed genes in anti-PD-1 datasets. **(E)** pathways identified in Treg genes and not collaborated with PD-1 signalling.

Recent report showed that non-overlapping roles of PD-1 and Foxp3 in maintaining immune tolerance, which was collaborated well with our findings (131). We hypothesized that PD-1 plays significant roles in promoting DEGs in Treg from normal tissues, non-tumor diseased tissues and tumor tissues. To test this hypothesis, we placed all the PD-1-induced Treg genes cells with Treg upregulated genes in the >1 log2 in the Y-axis, and anti-PD-1 antibody treated (PD-1 suppressed) genes in the >1 log2 in the X-axis (**Figure 8A**). The argument was that if gene expressions are promoted by PD-1, the genes will be shown in PD-1 induced gene groups; otherwise, they will shown in PD-1 suppressed gene group. If any genes upregulated or downregulated in tissue Treg, non-tumor diseased Treg and tumor Treg are not collaborated with PD-1 regulation, they will be placed in non-collaboration gene groups. As shown in **Figure 8B**, we found that there were a large numbers of PD-1 collaboration Treg genes upregulated and also significant numbers of PD-1 collaboration Treg genes downregulated in normal tissue Treg (14 – 163 genes upregulated; 3 – 48 genes downregulated). In addition, there were a large numbers of PD-1 collaboration Treg genes upregulated in non-tumor diseased tissue Treg (63-178 genes upregulated) and also significant numbers of PD-1 collaboration Treg genes downregulated (18 – 34 genes). Moreover, there were a number of PD-1 non-collaboration Treg genes upregulated in normal and non-tumor diseased tissue Treg (0 – 31 genes upregulated). Finally, there were a few numbers of PD-1 collaboration Treg genes upregulated in tumor tissue Treg (1 – 8 genes upregulated). We also found 12 pathways associated with PD-1 non-collaboration genes shared by Treg in various groups (**Figure 8E**); 9 pathways associated with PD-1 non-collaboration genes shared by Treg in normal tissues; 13 pathways associated with PD-1 non-collaboration genes shared by Treg in non-tumor diseased tissues; and 22 pathways with PD-1 non-collaboration genes shared by Treg in tumor tissues.

Our results have demonstrated that normal tissue Treg and non-tumor diseased tissue Treg have quite low CTLA-4 dependent but high PD-1 dependent transcriptomic changes, suggesting that CTLA-4 does not, but PD-1 does, play significant Foxp3-non-overlapped (131) roles in promoting Treg upregulated genes in normal and non-tumor diseased tissue Treg; and tumor spleen Treg and tumor Treg have certain transcriptomic changes in CTLA-4, and PD-1, non-collaboration manners with innate immune dominant pathways. Our results were correlated well with others reports on the effects of immune checkpoint receptors in Treg in pregnancy (132), Treg in autoimmunity (133), T cells in heart (134), and effects of immune checkpoint inhibitors on tumors (135–137).

### Tumor Tregs Downregulate More Immunometabolic and Trained Immune Metabolic Enzyme Genes Than Tregs From Other Groups

**Figure 9:**
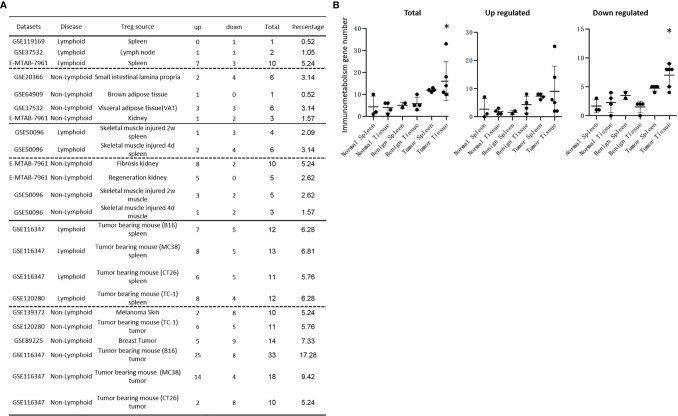
**(A)** Tumor tissue Treg groups upregulate more immunometabolism enzymes than other normal tissue Treg groups and benign disease tissue Treg groups. A total of 191 immunometabolism enzyme genes were screened in all the Treg groups. Downregulated and total immunometabolism gene numbers were more in tumor tissue Treg groups than in other Treg groups. **(B)** There were more differentially expressed immunometabolism enzyme genes in tumor tissue Treg groups than other Treg groups. The total regulated immunometabolism gene numbers were more in tumor tissue Treg groups than that in normal tissue Treg groups and diseased tissue Treg groups, *p* = 0.03. The upregulated immunometabolism gene numbers had no difference between Treg groups. The downregulated immunometabolism enzyme genes were more in tumor tissue Treg groups than other Treg groups, p < 0.05. *Significantly changed.

**Figure 10:**
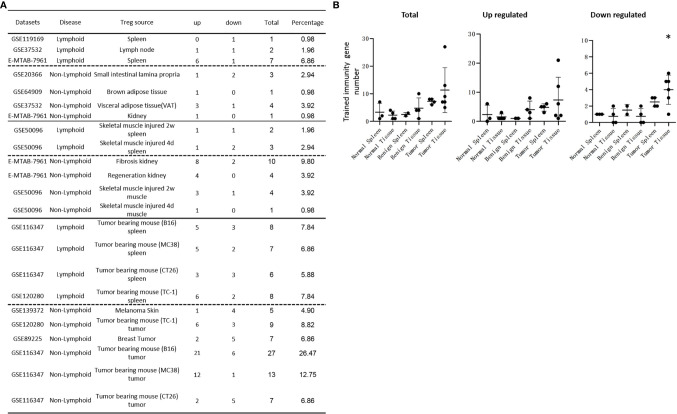
**(A)** Tumor tissue Treg groups upregulated more Trained immunity enzymes than normal tissue Treg groups and benign disease tissue Treg groups. The expressions of total of 102 trained immunity enzyme genes were screened in all the Treg groups. Upregulated Trained immunity enzyme genes in tumor spleen Treg groups were more than in the normal tissue Treg groups and benign disease tissue Treg groups. However, the differences were not statistically significant. Downregulated, and total Trained immunity enzyme genes were more in tumor tissue Treg groups than in other Treg groups. **(B)** There were more differentially expressed trained immunity enzyme genes in tumor tissue Treg groups than other Treg groups. The total regulated trained immunity enzyme genes were more in tumor tissue Treg groups than that in normal tissue Treg group and diseased tissue Treg groups, *p* = 0.06. The upregulated trained immunity gene numbers had no differences between Treg groups. The downregulated trained immunity genes in tumor tissue Treg groups were more than other Treg groups, p < 0.05.

It has been reported that while glycolysis fuels the biosynthetic and bioenergetic needs necessary for proliferation and migration of human Treg, suppressive capacity is mainly maintained by oxidative metabolism (138); that several metabolic pathways have been reported to be involved in immunometabolism (139) including glycolysis, fermentation, pentose phosphage pathway (PPP), tricarboxylic acid cycle (TCA)/mitochondrial electron transport chain (ETC), mitochondrial regulation, fatty acid metabolism, amino acid metabolism, and metabolic regulation and signaling; and that termed “trained immunity”, allows innate immune cells such as macrophages, monocytes, natural killer cells and endothelial cells (50) to shown enhanced responsiveness when they re-encounter pathogens and danger associated molecular patterns (140, 141), in which metabolic reprogrammings such as glycolysis, acetyl-CoA generation and mevalonate synthesis pathways (50, 51). We hypothesized that the expressions of immunometabolic enzyme genes can be found in Treg upregulated genes and downregulated genes in normal, non-tumor diseased and tumor tissues. To test this hypothesis, we collected 191 immunometabolic enzyme genes (50, 138, 139). As shown in **Figure 9A**, 0 – 7 genes, 1 – 8 genes and 2 – 25 immunometabolic enzyme genes were upregulated in normal tissue Treg, non-tumor diseased tissue Treg and tumor Treg, respectively. In addition, immunometabolic enzyme genes were downregulated more in tumor Treg than normal tissue Treg and non-tumor diseased tissue Treg (**Figure 9B**). When we zoomed in the expression changes of trained immunity metabolic enzyme genes in Treg, we found that, as shown in **Figure 10A**, trained immunity metabolic enzyme genes in Treg from fibrosis kidney (8 genes), mouse B16 tumor (21 genes) and mouse MC38 tumor (12 genes) were increased. In comparison, trained immunity metabolic enzyme genes in Treg from normal tissue Treg, non-tumor diseased tissue Treg and tumor Treg had a few genes upregulated. Once again, trained immunity metabolic enzyme genes were downregulated more in tumor Treg than normal tissue Treg and non-tumor diseased tissue Treg (**Figure 10B**). These results have demonstrated that the expressions of some trained immunity metabolic enzyme genes and global immunometabolic enzyme genes are downregulated in tumor Treg, suggesting that some innate immune metabolic pathways are suppressed in tumor Treg than normal tissue Treg and non-tumor diseased Treg.

### Reactive Oxygen Species (ROS) Regulate Treg Transcriptomes; and ROS-Suppressed Genes Are Downregulated More in Tumor Treg Than Treg From Other Groups

**Figure 11:**
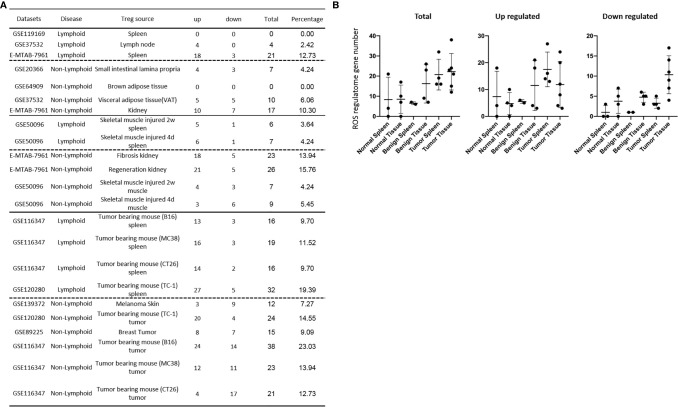
**(A)** Reactive oxygen species (ROS) regulatome DEGs in tumor tissue Treg groups were more than that in the normal tissue Treg and benign diseased tissue Treg groups. A total of 165 ROS regulatome genes (**Supplementary Table 5**) were screened in all the Treg groups. The upregulated ROS regulatome genes in tumor spleen Treg groups were more than that in the normal tissue Treg and benign diseased tissue Treg groups. However, the differences were not statistically significant. The downregulated ROS regulatome gene numbers were more in tumor tissue Treg groups than in other Treg groups **(B)** The differentially expressed ROS regultome genes in tumor tissue Treg groups were more than other Treg groups. The total regulated ROS regulatome gene numbers were more in tumor tissue Treg groups than that in normal tissue Treg groups, *p* = 0.16. The upregulated ROS regulatome gene numbers had no statistical differences between Treg groups. The downregulated ROS regulatome gene numbers were statistically more than other Treg groups, p < 0.05.

It has been reported that DDB1- and CUL4-associated factor 1 (DCAF1, a component of E3 ubiquitin-protein ligase complex) is downregulated in aged Tregs and is critical to restrain Treg aging *via* ROS regulated by glutathione (GSH)-S-transferase P (GSTP1). Importantly, interfering with GSTP1 and ROS pathways re-invigorates the proliferation and function of aged Tregs (142). Another report also showed that elimination of excessive ROS by treating Treg with ROS scavengers such as N-acetyl-L-cysteine (NAC) and GSH can rejuvenate the aged Treg and reinvigorate their suppressive function (143), suggesting that DCAF-regulated ROS is the culprit underlying Treg aging and aberrant function in immune senescence and inflammaging (143). TGF-β/SRY-Box Transcription Factor 4 (SOX4) axis-mediated upregulation of ecto-nucleoside triphosphate diphosphohydrolase (NTPDase, CD39) is counteracted by ROS-driven autophagy (144). We hypothesized that ROS regulatomes are regulated in tissue Treg, non-tumor diseased tissue Treg and tumor Treg. As shown in **Figure 11A**, the expressions of some of 165 ROS regulatomic genes were differentially regulated in tissue Treg. The upregulations of ROS regulatomic genes were found in LN Treg (4), splenic Treg (18), intestine Treg (4), BAT Treg (0), VAT Treg (5), kidney Treg (10), injured skeletal muscle (2 weeks) splenic Treg (5), injured skeletal muscle (4 days) splenic Treg (6), fibrosis kidney Treg (18), regeneration kidney Treg (21), injured skeletal muscle (2 weeks) Treg (4), injured skeletal muscle (4 days) Treg (3), B16 splenic Treg (13), MC38 splenic Treg (16), CT26 splenic Treg (14), TC-1 splenic Treg (27), melanoma Treg (3), TC-1 tumor Treg (20), breast tumor Treg (8), B16 tumor Treg (24), MC38 tumor Treg (12) and CT26 tumor Treg (4), respectively. As shown in **Figure 11B**, there were no statistical differences between Treg groups in upregulated ROS regulatomic genes. However, tumor tissue Treg groups significantly downregulated some ROS regulatomic genes, which may be responsible for increased Treg suppressive functions in Treg from tumors.

**Figure 12:**
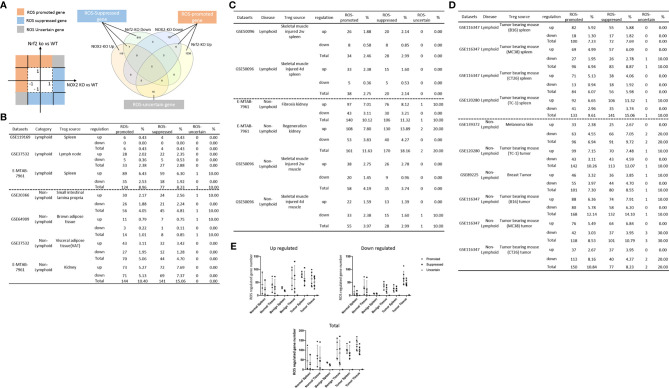
**(A)** A total of 2330 reactive oxygen species (ROS)-regulated genes were identified in the nicotinamide adenine dinucleotide phosphate (NADPH) oxidase oxidase 2 (NOX2) deficient microarray and anti-oxidant transcription factor Nuclear factor (erythroid-derived 2) factor 2 (NRF-2) deficient microarray datasets. All the ROS regulated genes were classified into 3 categories: *1)* 1384 ROS-promoted genes, *2)* 936 ROS suppressed gene, and *3)* 10 ROS regulation-uncertain genes. All the DEGs in all the Treg groups were examined in NOX2 deficiency-regulated genes (GSE100671) and Nrf2 (GSE7810) deficiency-regulated -datasets. **(B)** There were no differences of ROS regulated genes between Normal spleen Treg groups and normal tissue Treg groups. **(C)** The benign disease tissue Treg groups had more ROS-regulated genes than the benign diseased spleen Treg groups. Both ROS-promoted genes and ROS-suppressed gene numbers in benign diseased non-lymphoid tissue Treg groups were more than that of lymphoid tissue Treg groups, p < 0.05. **(D)** There were no differences of ROS-regulated genes between tumor spleen Treg groups and tumor tissue Treg groups. Tumor tissue Treg groups and tumor spleen Treg groups had no significant differences in both ROS-promoted genes and ROS-suppressed genes. **(E)** Tumor tissue Treg groups had more downregulated reactive oxygen species (ROS)-regulated genes than other Treg groups. The average numbers of upregulated ROS promoted genes were more in the benign diseased tissue Treg groups, tumor spleen Treg groups and tumor tissue Treg groups than other Treg groups. However, the differences were not statistically significant. The average numbers of downregulated ROS-suppressed genes were more in the tumor tissue Treg groups than other Treg groups p < 0.05.

Recently, we proposed a new concept that ROS are an integrated sensing system for metabolic homeostasis and alarming metabolic dangers (145). Nicotinamide adenine dinucleotide phosphate (NADPH) oxidase 2 (NOX2) inhibits the suppressive ability of Tregs by limiting NF-κB and Foxp3 activation (146). Dietary extra virgin olive oil attenuates kidney injury in pristane-induced SLE model *via* activation of HO-1/Nuclear factor (erythroid-derived 2)-like-2 (NRF2) antioxidant pathway and suppression of JAK/STAT, NF-κB and MAPK activation (147). Epigallocatechin-3-gallate prevents lupus nephritis development in mice *via* enhancing the NRF2 antioxidant pathway and inhibiting NLRP3 inflammasome activation (148). We then hypothesized that ROS modulate Treg transcriptomic genes. To examine this hypothesis and identify ROS promoted genes and ROS suppressed genes, in **Figure 12A**, we analyzed the catalytic, membrane-bound subunit of NOX2 KO microarray dataset (GSE100671) (146) and antioxidant transcription factor NRF2 KO microarray dataset (GSE7810). As we reported recently (28), in **Figure 12A**, if NOX2 KO upregulated genes were co-expressed with NRF2 KO-downregulated genes, these genes were classified as ROS promoted genes. If NOX2 KO downregulated genes were co-expressed with NRF2 KO upregulated genes, those genes were classified as ROS-suppressed genes. Among 2,330 genes identified in NOX2 KO and NRF2 KO, 1384 genes were ROS promoted genes whereas 936 genes were ROS-suppressed genes, and 10 genes were ROS uncertain genes (did not follow the classification). We found that Treg in normal tissues upregulated ROS promoted genes in the range of 6 – 89 genes. Treg in non-tumor diseased tissues upregulated ROS promoted genes in the range of 22 – 108 genes. Treg in tumor tissues upregulated ROS promoted genes in the range of 33 – 99 genes. As shown in **Figures 12B–D**, the average numbers of upregulated ROS-promoted genes were more in Treg from benign disease tissue, Treg from tumor spleens and tumor tissues than other groups, however, the differences were not statistically significant. The average numbers of downregulated ROS-suppressed genes were more in the Treg from tumor tissues than that of Treg from other groups, *p* < 0.05 (**Figure 12E**). These results suggest that ROS-suppressed immunosuppressive genes are downregulated more in tumor Treg than Treg from other groups.

## Discussion

Since Treg have a great therapeutic potential for numerous diseases (149) including cardiovascular disease (150), monogenic disease (immunodysregulation polyendocrinopathy enteropathy X-linked (or IPEX) syndrome), systemic lupus erythematosus, organ-specific autoimmune diseases (type I diabetes, psoriasis, myasthenia, inflammatory bowel disease, and multiple sclerosis), transplantation, and cancers (136, 149), Treg have been under intensified investigation continuously (69) since 1995 (151). Many wonderful technological advances including single-cell RNA sequencing (152) have been made to profile lymphoid and non-lymphoid tissue Treg heterogeneity. However, some important functional issues remained poorly characterized including upregulated signaling pathways in Treg from injured tissues and regenerated tissues, tumor splenic Treg specific pathways, tumor tissue Treg’s specific pathways, Treg canonical secretome, Treg non-canonical secretomes, Foxp3 collaboration/non-collaboration transcriptomes, immune checkpoint receptors PD-1 and CTLA-4 collaboration/non-collaboration transcriptomes, and ROS regulated Treg transcriptomes. To address those issues, we performed a comprehensive transcriptomic database mining with the strategies we pioneered (7, 28, 72, 153) to compare Tregs from normal lymphoid tissues and non-lymphoid tissues to Tregs from injured tissues and regenerated tissues, tumor splenic Tregs and tumor tissue Tregs. We made the following significant findings: *1)* Normal lymphoid Tregs have five specific (S-) pathways, diseased kidney Tregs have 17 S-pathways, and splenic Tregs from mouse with injured muscle have 3 S-pathways, suggesting that tissue injuries and diseases re-shape Treg transcriptomes; *2)* Tumor splenic Tregs share 12 pathways with tumor Tregs; tumor splenic Tregs have 11 S-pathways; and tumor Tregs have 8 S-pathways; *3)* 52 pathways are upregulated and 11 pathways are downregulated in Tregs from normal, and non-tumor disease tissues; *4)* Upregulated genes in tumor Tregs are lower than that in Tregs from normal tissues, non-tumor diseased tissues, and tumor spleens; tumor Tregs are different from Tregs from normal tissues, non-tumor diseased tissues and tumor spleens in 11 new innate immune pathways; 5) Normal and non-tumor disease Tregs upregulate canonical secretomic genes (2,641) with 24 pathways, share pathways such as p38 MAPK, TLR/IL-1, and IL-6 pathways; and tumor Tregs upregulate canonical secretomic genes in 17 pathways with tumor-Treg specific manners; 6) Normal tissue Tregs and non-tumor diseased tissue Tregs upregulate 56 exosome secretomic pathways (ESP), tumor Treg ESP are enriched with vitamin-C transport and endothelin-1 signaling, and are more focused than other Treg groups; 7) Normal tissue Tregs, non-tumor diseased tissue Tregs and tumor Tregs upregulate novel innate immune caspase-1 secretomic genes and innate immune caspase-4 secretomic genes to fulfill their tissue-specific, secretomes-specific functions and shared functions. This is the first report that Treg may use canonical and innate immune non-canonical secretomes including exosomes to fulfill their immunosuppressive and tissue regeneration functions; 8) Most tissue Treg transcriptomes are controlled by Foxp3; Tumor Tregs had significantly increased Foxp3 non-collaboration genes with ROS and 17 other pathways, suggesting that ROS and 17 other pathways may promote tumor Treg in increasing Foxp3 non-collaboration genes; 9) PD-1 does, but CTLA-4 does not, play significant roles in promoting Treg upregulated genes in normal and non-tumor diseased tissue Tregs; and tumor splenic and tumor Tregs have certain CTLA-4-, and PD-1-, non-collaboration transcriptomic changes with innate immune dominant pathways; 10) Tumor Tregs downregulate more immunometabolic and trained immune metabolic enzymes genes than Tregs from other groups; and 11) ROS significantly regulate Treg transcriptomes; and ROS-suppressed genes are downregulated more in tumor Treg than Treg from other groups.

Recently, we reported that while histone deacetylase 6 (HDAC6) and follicular Th cell-specific transcription factor B-cell lymphoma 6 (Bcl6) are important regulators of Treg plasticity, Th2-specific transcription factor GATA Binding Protein 3 (GATA3) determine the fate of plastic Treg by controlling whether it will convert in to either Th1-Treg or antigen-presenting cell (APC)-Treg (16); and Treg from spleen, lymph nodes, intestine and visceral adipose tissues promote tissue repair by generating secretomes similar to that of stem cells; and sharing TFs aryl hydrocarbon receptor (AHR), ETS variant transcription factor 5 (ETV5), early growth response 1 (EGR1), and Kruppel like factor 4 (KLF4) with stem cells, and Treg canonical secretomes and transcriptomes may be regulated by 1176 cytokines, 1706 canonical secretomes, kinome (complete list of human genome-encoded 651 kinases), cell surface receptors such as the complete list of 373 clusters of differentiation (CDs), the complete list of 1496 transcription factors, and 305 cell death regulatome (87). However, several important knowledge gaps presented in the INTRODUCTION remained unknown: 1) are there differences in Treg transcriptomes between Tregs from normal lymphoid and non-lymphoid tissues, injured tissues, regenerative tissues, tissues from mouse carrying tumor, tumor tissues; 2) are secretomes generated in Tregs in various tissues and pathological conditions; and 3) how Foxp3, immune checkpoint receptors CTLA-4 and PD-1 (19), immunometabolic pathways, trained immunity metabolic reprogramming (51, 141) and ROS pathways (145) regulate tissue Treg heterogeneity and Treg various secretomes.

**Figure 13:**
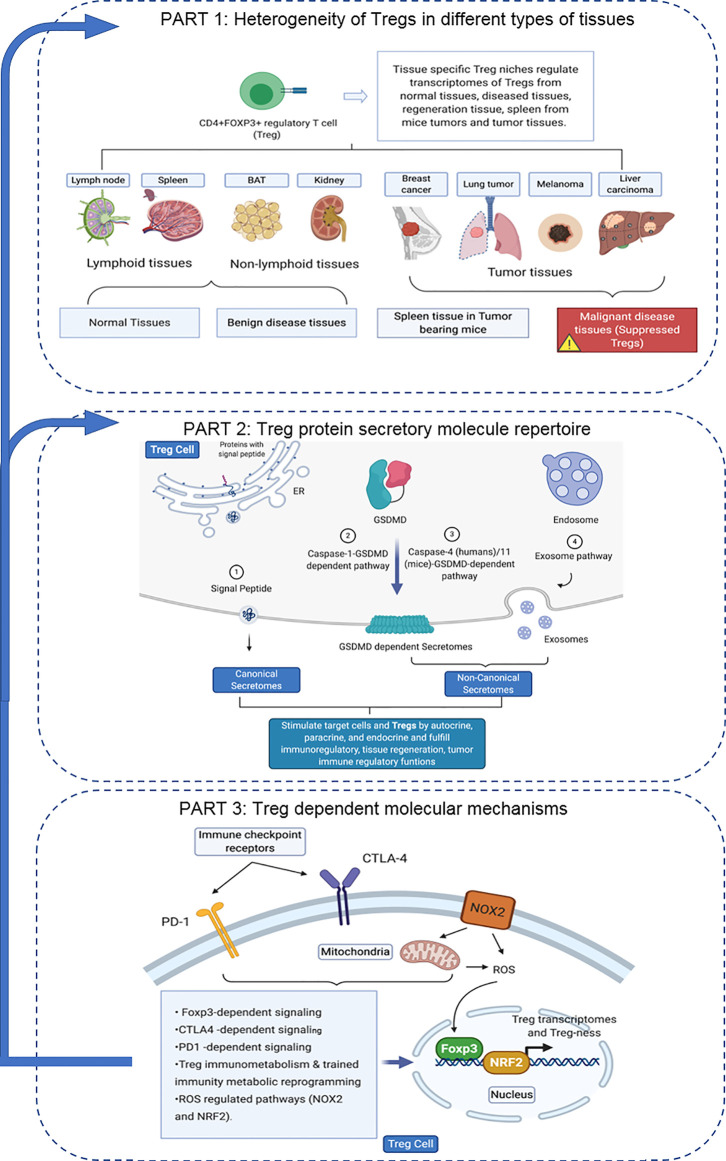
Canonical secretome, caspase-1-, caspase-4/11-gasdermin D non-canonical secretomes and exosomes may contribute to Treg heterogeneity, immunosuppression, tissue repair and weaken anti-tumor immune responses *via* ROS pathways, Foxp3, PD-1, CTLA-4, immunometabolism and trained immunity metabolic reprogramming. BAT, Brown adipose tissue; GSDMD, Gasdermin D; ER, Endoplasmic reticulum; PD-1, Programmed cell death protein 1; CTLA4, Cytotoxic T-lymphocyte-associated protein 4; ROS, Reactive oxygen species; Nox2, Nicotinamide adenine dinucleotide phosphate (NADPH) oxidase 2; Nrf2, The nuclear factor erythroid 2 (NFE2)-related factor 2; FOXP3, Forkhead box P3.

Based on our results, we propose a new working model. *First*, as shown in **Figure 13**, Tregs from normal lymphoid, non-lymphoid, injured muscle, fibrosis kidney, regeneration kidney, spleens from mouse with injured muscles and tumors, tumors have significant transcriptomic heterogeneity and signaling pathways; *Second*, in addition to immunosuppressive cytokines such as IL-10, TGF-β and IL-35, Treg may use much large secretomes such as canonical secretomes with proteins carrying signal peptide, non-canonical secretomes including caspase-1-GSDMD-dependent secretomes and innate immune caspase-4/11-GSDMD-dependent secretomes and exosome secretomes to act on target cells and Tregs themselves to maintain Treg niches for Treg heterogeneity and Treg-ness (87), fulfill immunosuppressive, tissue regeneration, tumor immunomodulatory functions; and *Third*, immunometabolism, innate immune memory (trained immunity) metabolic reprogramming, and ROS together with three well-characterized master regulators such as Treg transcription factor Foxp3, immune checkpoint receptors PD-1 and CTLA-4 regulate tissue transcriptomes and secretomes. Our findings have provided novel insights on tissue Treg niches for Treg heterogeneity and Treg-ness, and new therapeutic targets for immunosuppression, tissue repair, cardiovascular diseases, chronic kidney disease, autoimmune diseases, transplantation, and cancers.

One limitation of the current study is that due to the low throughput nature of verification techniques in the laboratories, we could not verify every result we identified with the analyses of high throughput data (see **Table 1** of Dr. Lai’s paper (1), and Table 10 of Dr. Zhang’s paper (28) for explanations). We acknowledge that carefully designed *in-vitro* and *in-vivo* experimental models will be needed to verify all the findings further and underlying mechanisms. Nevertheless, our findings provide novel insights on the roles of tissue Treg in controlling immune responses, and promoting tissue repair and regeneration as well as novel targets for the future therapeutic interventions for immunosuppression, cardiovascular diseases, autoimmune diseases, transplantation, cancers and tissue repair.

## Data Availability Statement

The original contributions presented in the study are included in the article/**Supplementary Material**. Further inquiries can be directed to the corresponding author.

## Author Contributions 

DN carried out the data gathering, data analysis and prepared tables and figures. TT, YL, KX, YiS, FS, JS, LL, CV, YuS, WH, JL-P, JL, XJ, EC, and HW aided with analysis of the data. XY supervised the experimental design, data analysis, and manuscript writing. All authors contributed to the article and approved the submitted version.

## Funding

DN was supported by the hospital fellowship.

## Conflict of Interest

The authors declare that the research was conducted in the absence of any commercial or financial relationships that could be construed as a potential conflict of interest.
