# Supplementary material for: Canonical Secretomes, Innate Immune Caspase-1-, 4/11-Gasdermin D Non-Canonical Secretomes and Exosomes May Contribute to Maintain Treg-Ness for Treg Immunosuppression, Tissue Repair and Modulate Anti-Tumor Immunity via ROS Pathways
Source: Front Immunol. 2021 May 18;12:678201. doi: 10.3389/fimmu.2021.678201 (PMC8168470; doi:10.3389/fimmu.2021.678201)
Supplement: Supplementary file 1 [file Presentation_1.pptx]

## Slide 1
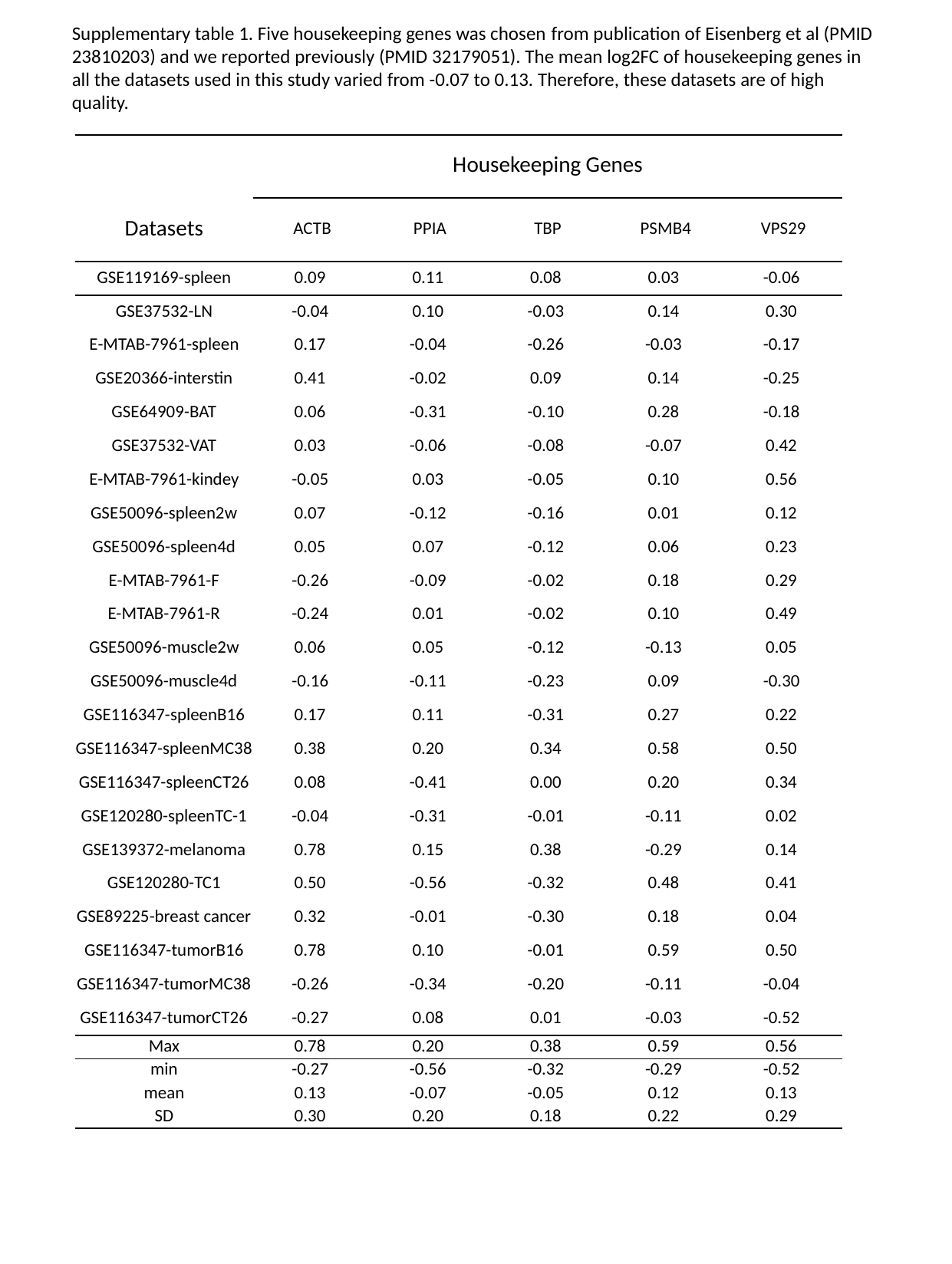

Supplementary table 1. Five housekeeping genes was chosen from publication of Eisenberg et al (PMID 23810203) and we reported previously (PMID 32179051). The mean log2FC of housekeeping genes in all the datasets used in this study varied from -0.07 to 0.13. Therefore, these datasets are of high quality.
| | | Housekeeping Genes | | | |
| --- | --- | --- | --- | --- | --- |
| Datasets | ACTB | PPIA | TBP | PSMB4 | VPS29 |
| GSE119169-spleen | 0.09 | 0.11 | 0.08 | 0.03 | -0.06 |
| GSE37532-LN | -0.04 | 0.10 | -0.03 | 0.14 | 0.30 |
| E-MTAB-7961-spleen | 0.17 | -0.04 | -0.26 | -0.03 | -0.17 |
| GSE20366-interstin | 0.41 | -0.02 | 0.09 | 0.14 | -0.25 |
| GSE64909-BAT | 0.06 | -0.31 | -0.10 | 0.28 | -0.18 |
| GSE37532-VAT | 0.03 | -0.06 | -0.08 | -0.07 | 0.42 |
| E-MTAB-7961-kindey | -0.05 | 0.03 | -0.05 | 0.10 | 0.56 |
| GSE50096-spleen2w | 0.07 | -0.12 | -0.16 | 0.01 | 0.12 |
| GSE50096-spleen4d | 0.05 | 0.07 | -0.12 | 0.06 | 0.23 |
| E-MTAB-7961-F | -0.26 | -0.09 | -0.02 | 0.18 | 0.29 |
| E-MTAB-7961-R | -0.24 | 0.01 | -0.02 | 0.10 | 0.49 |
| GSE50096-muscle2w | 0.06 | 0.05 | -0.12 | -0.13 | 0.05 |
| GSE50096-muscle4d | -0.16 | -0.11 | -0.23 | 0.09 | -0.30 |
| GSE116347-spleenB16 | 0.17 | 0.11 | -0.31 | 0.27 | 0.22 |
| GSE116347-spleenMC38 | 0.38 | 0.20 | 0.34 | 0.58 | 0.50 |
| GSE116347-spleenCT26 | 0.08 | -0.41 | 0.00 | 0.20 | 0.34 |
| GSE120280-spleenTC-1 | -0.04 | -0.31 | -0.01 | -0.11 | 0.02 |
| GSE139372-melanoma | 0.78 | 0.15 | 0.38 | -0.29 | 0.14 |
| GSE120280-TC1 | 0.50 | -0.56 | -0.32 | 0.48 | 0.41 |
| GSE89225-breast cancer | 0.32 | -0.01 | -0.30 | 0.18 | 0.04 |
| GSE116347-tumorB16 | 0.78 | 0.10 | -0.01 | 0.59 | 0.50 |
| GSE116347-tumorMC38 | -0.26 | -0.34 | -0.20 | -0.11 | -0.04 |
| GSE116347-tumorCT26 | -0.27 | 0.08 | 0.01 | -0.03 | -0.52 |
| Max | 0.78 | 0.20 | 0.38 | 0.59 | 0.56 |
| min | -0.27 | -0.56 | -0.32 | -0.29 | -0.52 |
| mean | 0.13 | -0.07 | -0.05 | 0.12 | 0.13 |
| SD | 0.30 | 0.20 | 0.18 | 0.22 | 0.29 |
