# Supplementary material for: Canonical Secretomes, Innate Immune Caspase-1-, 4/11-Gasdermin D Non-Canonical Secretomes and Exosomes May Contribute to Maintain Treg-Ness for Treg Immunosuppression, Tissue Repair and Modulate Anti-Tumor Immunity via ROS Pathways
Source: Front Immunol. 2021 May 18;12:678201. doi: 10.3389/fimmu.2021.678201 (PMC8168470; doi:10.3389/fimmu.2021.678201)
Supplement: Supplementary file 2 [file Presentation_2.pptx]

## Slide 1
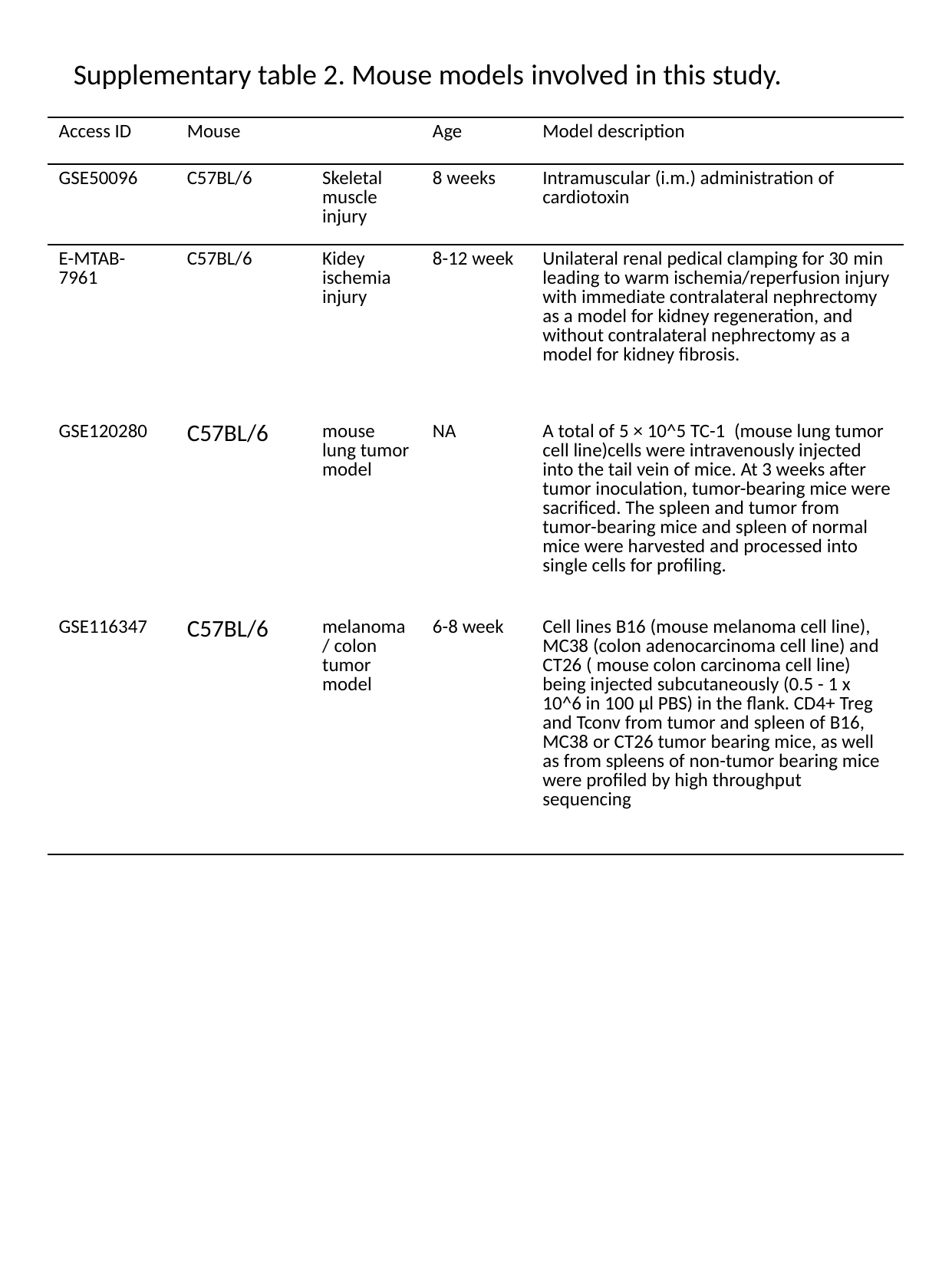

Supplementary table 2. Mouse models involved in this study.
| Access ID | Mouse | | Age | Model description |
| --- | --- | --- | --- | --- |
| GSE50096 | C57BL/6 | Skeletal muscle injury | 8 weeks | Intramuscular (i.m.) administration of cardiotoxin |
| E-MTAB-7961 | C57BL/6 | Kidey ischemia injury | 8-12 week | Unilateral renal pedical clamping for 30 min leading to warm ischemia/reperfusion injury with immediate contralateral nephrectomy as a model for kidney regeneration, and without contralateral nephrectomy as a model for kidney fibrosis. |
| GSE120280 | C57BL/6 | mouse lung tumor model | NA | A total of 5 × 10^5 TC-1 (mouse lung tumor cell line)cells were intravenously injected into the tail vein of mice. At 3 weeks after tumor inoculation, tumor-bearing mice were sacrificed. The spleen and tumor from tumor-bearing mice and spleen of normal mice were harvested and processed into single cells for profiling. |
| GSE116347 | C57BL/6 | melanoma/ colon tumor model | 6-8 week | Cell lines B16 (mouse melanoma cell line), MC38 (colon adenocarcinoma cell line) and CT26 ( mouse colon carcinoma cell line) being injected subcutaneously (0.5 - 1 x 10^6 in 100 μl PBS) in the flank. CD4+ Treg and Tconv from tumor and spleen of B16, MC38 or CT26 tumor bearing mice, as well as from spleens of non-tumor bearing mice were profiled by high throughput sequencing |
